# ChatGPT-Generated Differential Diagnosis Lists for Complex Case–Derived Clinical Vignettes: Diagnostic Accuracy Evaluation

**DOI:** 10.2196/48808

**Published:** 2023-10-09

**Authors:** Takanobu Hirosawa, Ren Kawamura, Yukinori Harada, Kazuya Mizuta, Kazuki Tokumasu, Yuki Kaji, Tomoharu Suzuki, Taro Shimizu

**Affiliations:** 1 Department of Diagnostic and Generalist Medicine Dokkyo Medical University Tochigi Japan; 2 Department of General Medicine Okayama University Graduate School of Medicine, Dentistry and Pharmaceutical Sciences Okayama Japan; 3 Department of General Medicine International University of Health and Welfare Narita Hospital Chiba Japan; 4 Department of Hospital Medicine Urasoe General Hospital Okinawa Japan

**Keywords:** artificial intelligence, AI chatbot, ChatGPT, large language models, clinical decision support, natural language processing, diagnostic excellence, language model, vignette, case study, diagnostic, accuracy, decision support, diagnosis

## Abstract

**Background:**

The diagnostic accuracy of differential diagnoses generated by artificial intelligence chatbots, including ChatGPT models, for complex clinical vignettes derived from general internal medicine (GIM) department case reports is unknown.

**Objective:**

This study aims to evaluate the accuracy of the differential diagnosis lists generated by both third-generation ChatGPT (ChatGPT-3.5) and fourth-generation ChatGPT (ChatGPT-4) by using case vignettes from case reports published by the Department of GIM of Dokkyo Medical University Hospital, Japan.

**Methods:**

We searched PubMed for case reports. Upon identification, physicians selected diagnostic cases, determined the final diagnosis, and displayed them into clinical vignettes. Physicians typed the determined text with the clinical vignettes in the ChatGPT-3.5 and ChatGPT-4 prompts to generate the top 10 differential diagnoses. The ChatGPT models were not specially trained or further reinforced for this task. Three GIM physicians from other medical institutions created differential diagnosis lists by reading the same clinical vignettes. We measured the rate of correct diagnosis within the top 10 differential diagnosis lists, top 5 differential diagnosis lists, and the top diagnosis.

**Results:**

In total, 52 case reports were analyzed. The rates of correct diagnosis by ChatGPT-4 within the top 10 differential diagnosis lists, top 5 differential diagnosis lists, and top diagnosis were 83% (43/52), 81% (42/52), and 60% (31/52), respectively. The rates of correct diagnosis by ChatGPT-3.5 within the top 10 differential diagnosis lists, top 5 differential diagnosis lists, and top diagnosis were 73% (38/52), 65% (34/52), and 42% (22/52), respectively. The rates of correct diagnosis by ChatGPT-4 were comparable to those by physicians within the top 10 (43/52, 83% vs 39/52, 75%, respectively; *P*=.47) and within the top 5 (42/52, 81% vs 35/52, 67%, respectively; *P*=.18) differential diagnosis lists and top diagnosis (31/52, 60% vs 26/52, 50%, respectively; *P*=.43) although the difference was not significant. The ChatGPT models’ diagnostic accuracy did not significantly vary based on open access status or the publication date (before 2011 vs 2022).

**Conclusions:**

This study demonstrates the potential diagnostic accuracy of differential diagnosis lists generated using ChatGPT-3.5 and ChatGPT-4 for complex clinical vignettes from case reports published by the GIM department. The rate of correct diagnoses within the top 10 and top 5 differential diagnosis lists generated by ChatGPT-4 exceeds 80%. Although derived from a limited data set of case reports from a single department, our findings highlight the potential utility of ChatGPT-4 as a supplementary tool for physicians, particularly for those affiliated with the GIM department. Further investigations should explore the diagnostic accuracy of ChatGPT by using distinct case materials beyond its training data. Such efforts will provide a comprehensive insight into the role of artificial intelligence in enhancing clinical decision-making.

## Introduction

### Decision-Making in Health Care

In health care, accurate diagnosis plays a critical role in the effective management of patients’ conditions [1]. Clinicians often rely on their expertise and various case presentations to make clinical decisions. However, the increasing complexity of cases, particularly those requiring referrals to specialized departments such as general internal medicine (GIM), and the rapid expansion of medical knowledge necessitate enhanced diagnostic support. A single-center study reported diagnostic error rates of 2% in an outpatient GIM department [2], while a systematic review found that the error rates exceeded by 10% in older adult patients [3]. Such inaccuracies underline the pressing need for tools to aid physicians in making more accurate diagnoses [4]. One promising avenue being explored is the application of clinical decision support (CDS) systems.

### CDS Tools

Various CDS systems, including symptom checkers [5] and differential diagnosis generators [6], have been developed over the years. The former are generally designed for the general public, while the latter are intended for health care providers. The journey of computer-aided health care traces back to the early 1970s, marked by a strong interest in harnessing computing power to enhance care quality. Historically, CDS tools often employ multistep processes that combine logical or computational processes, probability assessments, and heuristic methods. Notably, a combination of algorithms and heuristic rules has been integral to many medical applications [7]. There is evidence of CDS tools being utilized in the outpatient department of GIM [8]. However, despite the potential of CDS systems to boost diagnostic accuracy and efficiency, they often increase clinicians’ workload [9], particularly due to the need for structured input data. This remains a great barrier to their widespread adoption. In this context, artificial intelligence (AI), especially large language models, provides an alternative approach for health care support [10], particularly through the AI chatbot [11].

### ChatGPT in Health Care

AI chatbots such as ChatGPT have demonstrated potential in facilitating effective communication between patients and health care providers [12] and transforming medical writing [13]. ChatGPT, developed by OpenAI, is an application of large language model based on natural language processing, known as a generative pretrained transformer (GPT) [14]. It can generate human-like responses to user prompts. With the progression from the third-generation GPT (GPT-3.5) to the fourth-generation GPT (GPT-4), the model’s accuracy has improved in professional examinations [15] and multiple-choice problems across various languages [16]. Yet, AI chatbots are not exempt from limitations and risks [17,18]. These limitations encompass transparency issues [19], nonspecialized medical knowledge, outdated medical information, inherent biases, and a potential to disseminate misinformation [11]. Despite these challenges, AI systems such as ChatGPT are continually improving and hold promise as essential tools for achieving diagnostic excellence [20].

To prepare for potential clinical applications of AI chatbots, it is essential to evaluate their diagnostic accuracy, particularly for complex cases that frequently necessitate referral to specialized departments such as the GIM department. If harnessed correctly, generative AI like ChatGPT could reduce the diagnostic errors attributed to the inherent complexity of the GIM domain. This would streamline the department’s workflow, enhancing patient care and outcomes. The study will reveal the potential of generative AIs, including ChatGPT as the CDS, especially in the GIM department.

Previous studies have reported that the diagnostic accuracy of the differential diagnosis lists generated by ChatGPT for clinical vignettes falls between 64% and 83% [21,22]. A clinical vignette is a concise narrative used in research to present a clinical scenario. However, these earlier studies did not focus on the materials derived from the GIM department, which is known for its diagnostically challenging cases. This gap in the literature accentuates the novelty and distinctiveness of our study. We aimed to evaluate the diagnostic accuracy of the differential diagnosis lists generated by ChatGPT, specifically using clinical vignettes derived from case reports published by the GIM department. By focusing on these GIM case reports, our research potentially offers a more rigorous appraisal of the diagnostic prowess of ChatGPT compared to preceding studies. In line with this, we expect ChatGPT-4 to provide the correct diagnosis in its differential diagnosis lists with an accuracy consistent with or within the previously reported range of 64%-83%.

## Methods

### Study Design

We evaluated the diagnostic accuracy of the differential diagnosis lists generated by ChatGPT-3.5 and ChatGPT-4 for clinical vignettes from case reports published by the Department of GIM. The term “differential diagnosis” refers to a list of possible conditions or diseases that could be causing a patient’s symptoms and signs. It is created by considering the patient’s clinical history, physical examination, and the results of any investigations, thus aiding in the diagnostic process. This study was conducted at the GIM Department (Department of Diagnostic and Generalist Medicine) of Dokkyo Medical University Hospital, Shimotsuga, Tochigi, Japan.

### Ethical Considerations

Because this study used case vignettes from published case reports, approval by the ethics committee and requirement for individual consent were not required.

### Clinical Vignettes

We used clinical vignettes from case reports published by the GIM Department of Dokkyo Medical University Hospital. Clinical cases that were challenging to diagnose and typically involved a high level of complexity were often referred to the GIM department. Some of these cases were published as case reports in medical journals. To find case reports published in English from our department, we searched PubMed using the following keywords on March 20, 2023: “(Dokkyo Medical University [affil]) AND (Generalist Medicine [affil]) AND (2016/4/1:2022/12/31 [dp]) AND (Case Reports [PT]).” After finding 54 case reports in PubMed, 2 experienced GIM physicians (TH and RK) checked these case reports for diagnostic or nondiagnostic cases, assessed the final diagnosis, and displayed them as clinical vignettes. Two cases were excluded because they were nondiagnostic. In total, 52 cases were included in this study. For example, consider the case reports titled “Hepatic portal venous gas after diving” [23], which is mentioned as case number 3 in Table S1 of Multimedia Appendix 1 and Table S2 of Multimedia Appendix 2. From this report, we extracted the clinical vignette from the case description section: “A 68-year-old man with diabetes and...There was no evidence of pneumatosis intestinalis.” Decompression sickness was determined as the final diagnosis for this case. These case reports meet the standards required for publication in peer-reviewed journals and have been written and selected by experienced GIM physicians. Each clinical vignette included the clinical history, physical examination, and results of the investigation. The title, abstract, introduction, clinical assessment, differential diagnosis, final diagnosis, figures, legends, tables, and case reports were removed from the vignettes. The final diagnosis for each case, which had been established through the usual diagnostic processes and subsequently published in these case reports, was assessed and displayed in the form of clinical vignettes. The final diagnosis was confirmed by 2 experienced GIM physicians. Discrepancies between the 2 physicians were resolved through discussions. We also assessed the publication date and status of the included case reports as open access.

### Differential Diagnosis Lists Created by Physicians

The differential diagnosis lists for each clinical vignette were independently created by 3 other GIM physicians (KT, YK, and T Suzuki) not affiliated with Dokkyo Medical University. Each clinical vignette was allocated to 1 physician, resulting in an average of 17 case descriptions being handled by each physician. They were instructed to create the top 10 differential diagnosis lists in English by reading the same clinical vignettes, without consulting other physicians or using CDS tools. It is essential to highlight that the physicians did not adhere to any specific guidelines, criteria, or protocols during this process. They operated based solely on their expertise and experience. Before creating the differential diagnosis lists, they were confirmed to be unaware of the case reports, clinical vignettes, final diagnosis, and differential diagnosis lists generated by ChatGPT-3.5 and ChatGPT-4. The physicians also remained blinded to each other’s assessments. A computer-generated order table determined the sequence in which the clinical vignettes were presented.

### Differential Diagnosis Lists Generated by ChatGPT

We used ChatGPT, an application of the GPT-3.5 model (March 14 version; ChatGPT-3.5, OpenAI, LLC), on March 20, 2023. We also used ChatGPT, an application of the GPT-4 model (March 23 version; ChatGPT-4, OpenAI, LLC), on April 10, 2023. Neither of the ChatGPT models were specially trained or reinforced for medical diagnoses. The physician (TH) typed the following text in the prompt: “Tell me the top 10 suspected illnesses for the following symptoms: (copy and paste each clinical vignette).” The prompt was designed to encourage the ChatGPT models to generate a list of differential diagnoses. The rationale behind selecting this particular prompt was grounded in preliminary testing. In these tests, various prompts were evaluated for their effectiveness in soliciting a comprehensive list of potential illnesses. This prompt consistently yielded reliable and inclusive differential diagnoses in our initial evaluations.

To minimize potential bias, the order in which the vignettes were presented to ChatGPT-3.5 and ChatGPT-4 was determined using a computer-generated order table. To ensure no interference from previous responses, physicians cleared the previous conversation before introducing new clinical vignettes. We used the initial answers as the top 10 differential diagnosis lists generated by ChatGPT-3.5 and ChatGPT-4.

### Evaluation of Differential Diagnosis Lists

**Figure 1:**
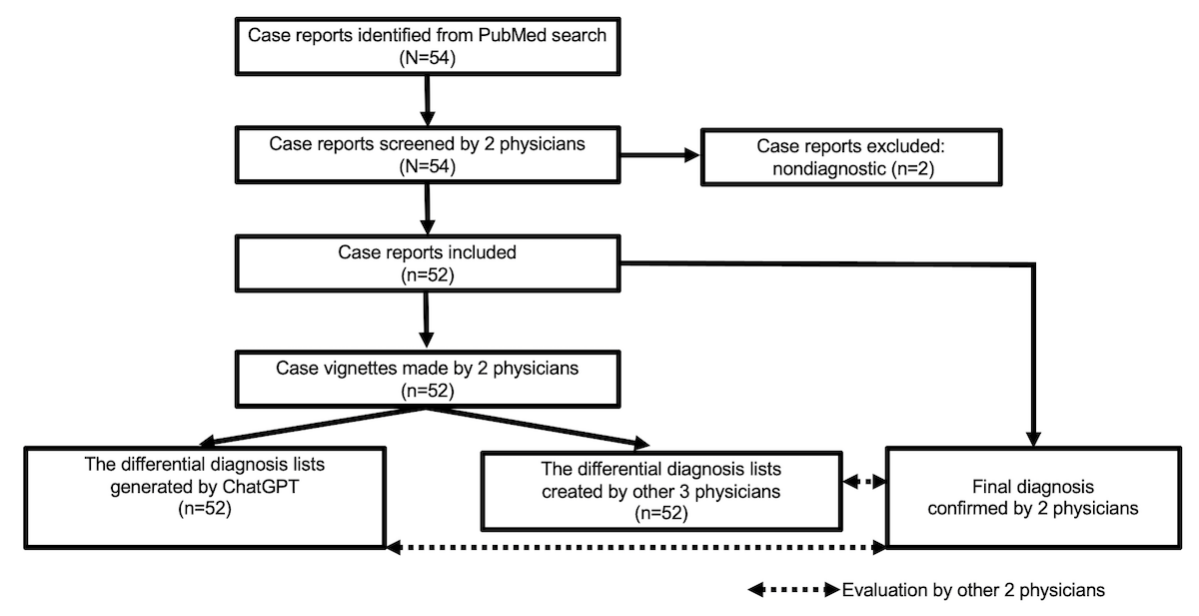
Study design.

**Figure 2:**
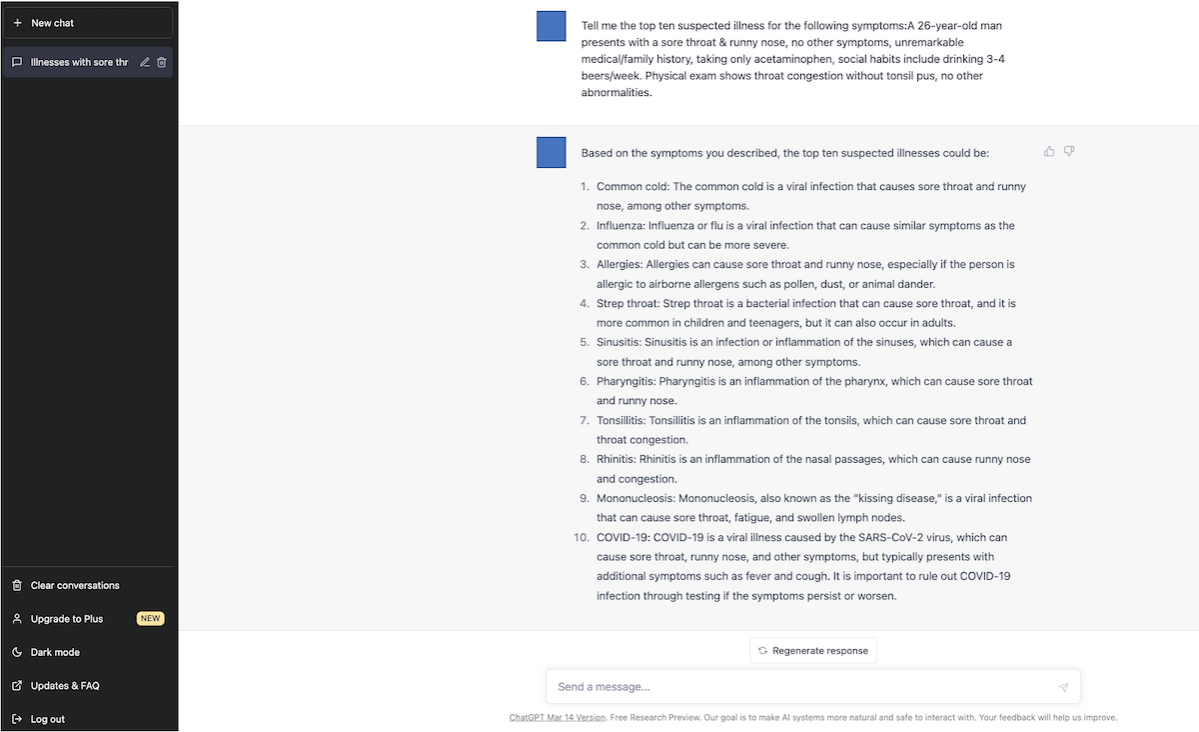
A differential diagnosis list generated by the third-generation ChatGPT for a sample case.

**Figure 3:**
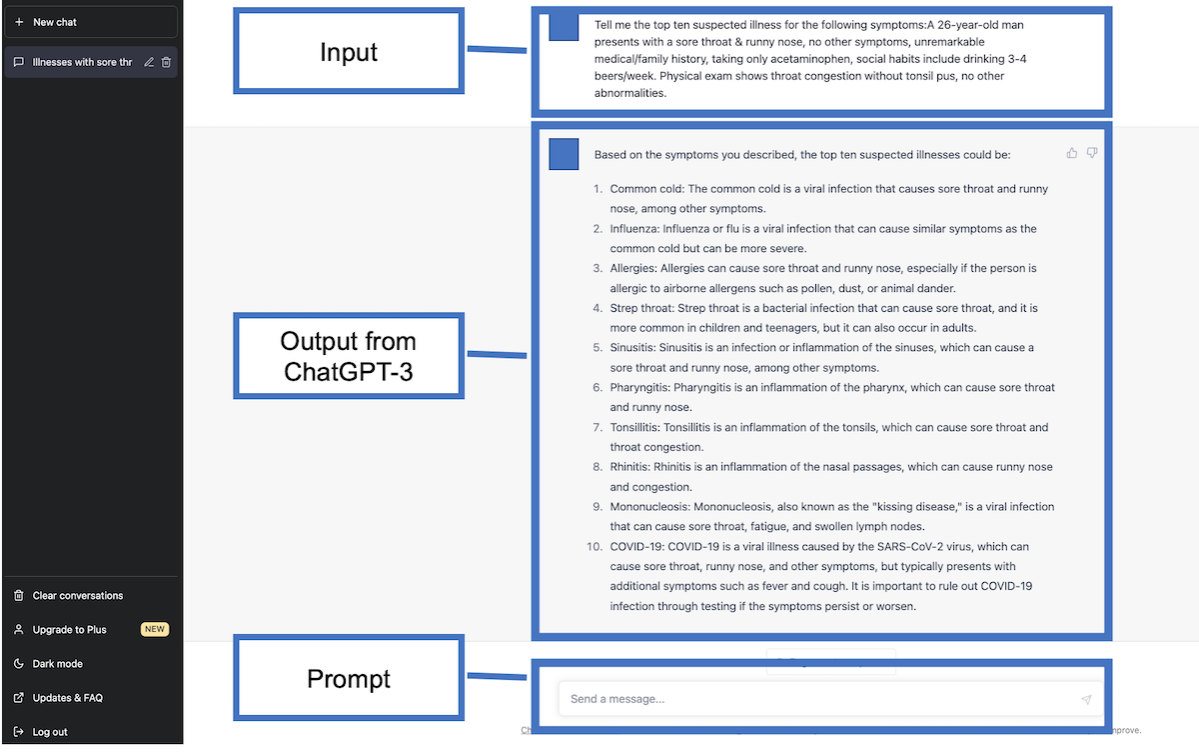
Explanation for the differential diagnosis list generated by the third-generation ChatGPT for a sample case. ChatGPT-3: third-generation ChatGPT.

**Figure 4:**
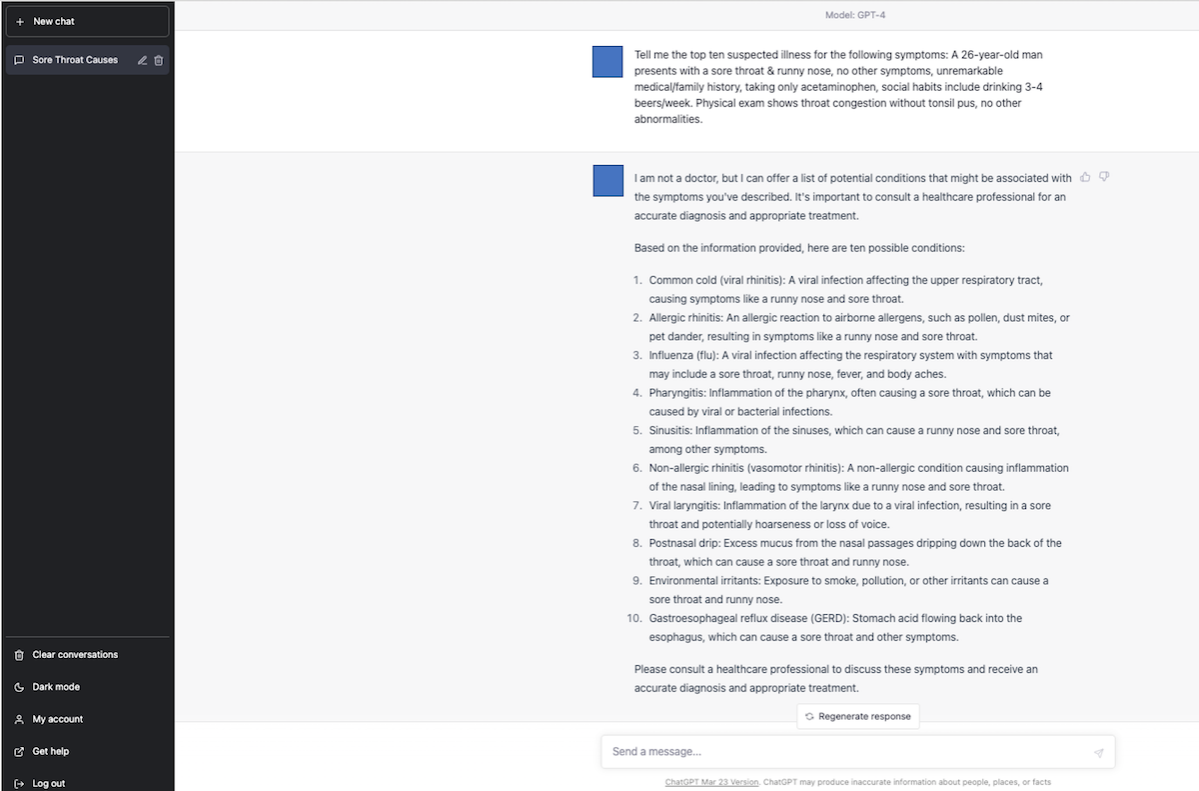
A differential diagnosis list generated by the fourth-generation ChatGPT for a sample case.

**Figure 5:**
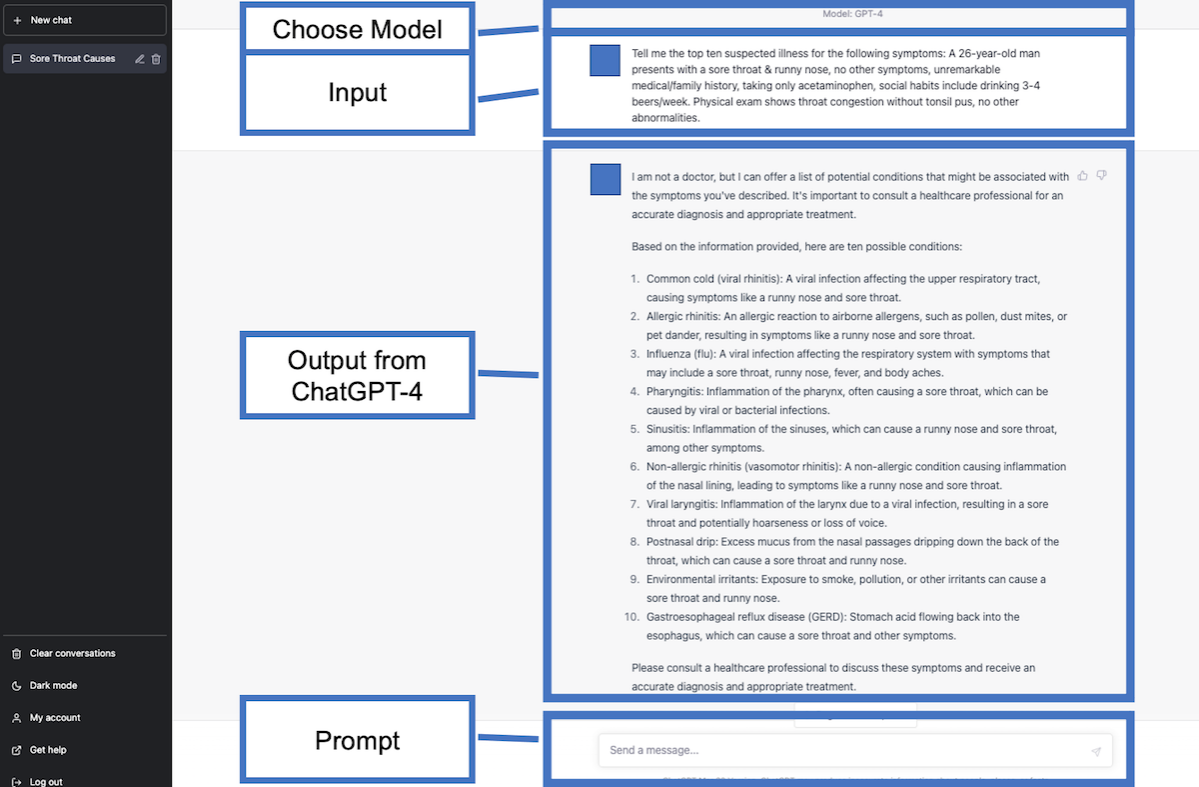
Explanation for the differential diagnosis list generated by the fourth-generation ChatGPT for a sample case. ChatGPT4: fourth-generation ChatGPT.

Two other GIM physicians (YH and KM) evaluated whether the final diagnosis was included in the differential diagnosis lists created by the physicians and those generated by ChatGPT models. A diagnosis was labeled “1” if it accurately and specifically identified the condition or was sufficiently close to the exact diagnosis that it would enable prompt and appropriate treatment. Conversely, a diagnosis was marked as “0” if it diverged significantly from the actual diagnosis [24]. When the final diagnosis was present, the researcher further assessed its ranking within the list. Discrepancies between the 2 evaluators were resolved through discussions. The study design is illustrated in Figure 1. Examples of a differential diagnosis list generated by ChatGPT-3.5 and ChatGPT-4 are shown in Figures 2-3 and Figures 4-5, respectively.

### Measurements

We measured the rate of correct diagnoses within the top 10 differential diagnosis lists, top 5 differential diagnosis lists, and top diagnosis provided by ChatGPT-3.5, ChatGPT-4, and the physicians. As a binary approach, we scored the presence of the final diagnosis on the list as one and its absence as zero. For an exploratory analysis, we compared the rates of correct diagnoses in the lists generated by ChatGPT-3.5 and ChatGPT-4 between case reports that were open access and those that were not. This comparison was motivated by understanding that GPT-3.5 and GPT-4 were primarily learned from open sources available on the internet [16]. Given that these models are predominantly trained on openly accessible data, we postulated that open access case reports might yield better diagnostic results than non–open access ones. Additionally, we compared the rates of correct diagnoses within the lists generated by ChatGPT-3.5 and ChatGPT-4 based on the publishing year prior to 2021 or in 2022. This distinction arises from the knowledge cutoffs for ChatGPT-3.5 and ChatGPT-4, which were set in early 2021. Since the models would be more familiar with data before this time and less informed about subsequent publications, we hypothesized that the case reports published in the years prior to 2021 could produce better diagnostic results than those published in 2022. However, the details of the learning data source and cutoff timing were not available to the public.

### Analysis

Categorical or binary variables were presented as numbers (percentages) and compared using the chi-square test. To mitigate the increased risk of type I error arising from multiple comparisons, we employed the Bonferroni correction [25]. Although alternative methods exist, we chose the Bonferroni correction for its strict control over false positives. When conducting multiple comparisons, we set the Bonferroni-corrected significance level at a *P* value <.02. This was derived by dividing .05 (the standard level of significance) by 3 (the number of comparisons undertaken). Both the chi-square test and the computation of the Bonferroni-corrected significance level were conducted in R (version 4.2.2; R Foundation for Statistical Computing) using the stats library (version 4.2.2).

## Results

### Case Report Profiles

In total, 52 case reports were included in this study, among which 39 (75%) were open access case reports. A total of 24 (46%) case reports were published prior to 2021. Of the total case reports, 12 (23%) were published in 2021 and 16 (31%) were published in 2022. The included case reports are presented in Multimedia Appendix 1.

### Diagnostic Performance

Representative examples of differential diagnosis lists with the final diagnosis are shown in Table 1.

The rates of correct diagnosis by ChatGPT-4 within the top 10 differential diagnoses, top 5 differential diagnoses, and top diagnosis were 83% (43/52), 81% (42/52), and 60% (31/52), respectively (Table 2). The rates of correct diagnosis by ChatGPT-3.5 within the top 10 differential diagnoses, top 5 differential diagnoses, and top diagnosis were 73% (38/52), 65% (34/52), and 42% (22/52), respectively. The rates of correct diagnosis by ChatGPT-4 were comparable to those by ChatGPT-3.5 within the top 10 (43/52, 83% vs 38/52, 73%, respectively; *P*=.34) and top 5 (42/52, 81% vs 34/52, 65%, respectively; *P*=.12) differential diagnosis lists and top diagnosis (31/52, 60% vs 22/52, 42%, respectively; *P*=.12), although the difference was not statistically significant. The rates of correct diagnosis by ChatGPT-4 were also comparable to those by physicians within the top 10 (43/52, 83% vs 39/52, 75%, respectively; *P*=.47) and top 5 (42/52, 81% vs 35/52, 67%, respectively; *P*=.18) differential diagnoses and top diagnosis (31/52, 60% vs 26/52, 50%, respectively; *P*=.43), although the difference was not statistically significant. Multimedia Appendix 2 shows the final diagnosis and the 10 differential diagnosis lists generated by ChatGPT-3.5 and ChatGPT-4 and those created by physicians.

Regarding open access case reports (Table 3), there were no statistically significant differences in the rates of correct diagnoses within the top 10 and top 5 differential diagnosis lists and top diagnoses generated by ChatGPT-3.5 and ChatGPT-4. Regarding the timing of publication, there were no statistically significant differences in ChatGPT-3.5 and ChatGPT-4 diagnoses between those published prior to 2021 and those published in 2022 (Table 3).

**Table 1:** Representative examples of differential diagnosis lists with the final diagnosis.

Final diagnosis	Differential diagnosis lists		
	ChatGPT-3.5^a^	ChatGPT-4^b^	Physicians
Tarsal tunnel syndrome	Peripheral neuropathy Tarsal tunnel syndrome Lumbar spinal stenosis Diabetes Alcohol abuse Vitamin B12 deficiency Hypothyroidism Chronic kidney disease Multiple sclerosis Charcot-Marie-Tooth disease	Peripheral neuropathy Tarsal tunnel syndrome Lumbar radiculopathy Small fiber neuropathy Chronic inflammatory demyelinating polyneuropathy Complex regional pain syndrome Peripheral artery disease Charcot-Marie-Tooth disease Polyneuropathy Leprosy	Sarcoidosis Chronic inflammatory demyelinating polyradiculoneuropathy Diabetic neuropathy Vitamin B12 deficiency Alcoholic neuropathy Paraneoplastic syndrome Antimyelin oligodendrocyte glycoprotein antibody-associated disease antineutrophil cytoplasmic antibody-related vasculitis POEMS (polyneuropathy, organomegaly, endocrinopathy, monoclonal plasma cell disorder, skin changes) syndrome Restless legs syndrome
Nonepisodic angioedema associated with eosinophilia	Eosinophilic fasciitis Acute interstitial nephritis Hypereosinophilic syndrome Churg-Strauss syndrome Idiopathic hypereosinophilia syndrome Lymphoma Systemic lupus erythematosus Sarcoidosis Infectious mononucleosis Allergic reactions	Eosinophilic cellulitis (Wells syndrome) Idiopathic eosinophilic oedema Hypereosinophilic syndrome Eosinophilic granulomatosis with polyangiitis (Churg-Strauss syndrome) Drug-induced eosinophilia Eosinophilic fasciitis (Shulman syndrome) Kimura disease Eosinophilic gastroenteritis Parasitic infections Systemic lupus erythematosus	Hypereosinophilic syndrome Idiopathic peripheral edema Idiopathic angioedema Cholinergic urticaria Eosinophilic granulomatosis with polyangiitis Parasitic infection Chronic eosinophilic leukemia Drug-induced (including supplements, herbal medicine) hypereosinphilic edema Tuberculosis Viral infection
Decompression sickness	Arterial gas embolism Decompression sickness Carbon monoxide poisoning Nitrogen narcosis Hypothermia Acute pancreatitis Mesenteric ischemia Gastroenteritis Gastric ulcer Acute cholecystitis	Decompression sickness Gas embolism Acute coronary syndrome Gastroenteritis Acute pancreatitis Peptic ulcer disease Diabetic ketoacidosis Acute cholecystitis Mesenteric ischemia Gastritis	Air embolization Decompression sickness Nonocclusive mesenteric ischemia Diabetic ketoacidosis Bacteremia Bacterial translocation Acute coronary syndrome Cholelithiasis Cholangitis Cholesterol embolization

^a^ChatGPT-3.5: third-generation ChatGPT.

^b^ChatGPT-4: fourth-generation ChatGPT.

**Table 2:** Rates of correct diagnoses within the top 10 and top 5 differential diagnosis lists and top diagnosis generated by ChatGPT-3.5 and ChatGPT-4 compared with those created by physicians.

Variable	ChatGPT-4^a^ (n=52), n (%)	ChatGPT-3.5^b^ (n=52), n (%)	Physicians (n=52), n (%)	*P* value^c^		
				ChatGPT-4 vs physicians	ChatGPT-3.5 vs physicians	ChatGPT-4 vs ChatGPT-3.5
Within the top 10	43 (83)	38 (73)	39 (75)	.47	>.99	.34
Within the top 5	42 (81)	34 (65)	35 (67)	.18	>.99	.12
Top diagnosis	31 (60)	22 (42)	26 (50)	.43	.56	.12

^a^ChatGPT-4: fourth-generation ChatGPT.

^b^ChatGPT-3.5: third-generation ChatGPT.

^c^*P* values from chi-square scores.

**Table 3:** Rates of correct diagnoses within the top 10 and top 5 differential diagnosis lists and top diagnosis generated by third-generation ChatGPT and fourth-generation ChatGPT between open access and non–open access case reports and between the timing of publications prior to 2021 and published in 2022.

Variable	Fourth-generation ChatGPT							Third-generation ChatGPT					
	Open access (n=39), n (%)	Non–open access (n=13), n (%)	*P* value^a^	Prior to 2021 (n=24), n (%)	In 2022 (n=16), n (%)	*P* value^b^	Open access (n=39), n (%)		Non–open access (n=13), n (%)	*P* value^a^	Prior to 2021 (n=24), n (%)	In 2022 (n=16), n (%)	*P* value^b^
Within the top 10	32 (82)	11 (85)	>.99	20 (83)	13 (81)	>.99	28 (72)		10 (77)	>.99	17 (71)	13 (81)	.71
Within the top 5	31 (80)	11 (85)	>.99	19 (79)	13 (81)	>.99	25 (64)		9 (69)	>.99	17 (71)	11 (69)	>.99
Top diagnosis	22 (56)	9 (69)	.62	17 (71)	9 (56)	.54	14 (36)		8 (62)	.19	11 (46)	8 (50)	>.99

^a^*P* values from chi-square scores comparing open access and non–open access case reports.

^b^*P* values from chi-square scores comparing between case reports prior to 2021 and case reports published in 2022.

## Discussion

### Principal Results

This study has several main findings. First, our study demonstrates the accuracy of the differential diagnosis lists generated by ChatGPT-3.5 and ChatGPT-4 for complex clinical vignettes from case reports. The rate of correct diagnoses within the top 10 and top 5 differential diagnosis lists generated by ChatGPT-4 was >80%. With a diagnostic accuracy of >80%, ChatGPT-4 can serve as a supplementary tool for physicians, especially when dealing with complex cases. Our results have demonstrated that GPT possesses diagnostic capabilities that can be comparable to those of physicians. This suggests that GPT might serve as a form of collective intelligence, capable of double-checking clinical diagnoses conducted by medical practitioners, at the very least. Second, there were no statistically significant differences in the rates of correct diagnoses by ChatGPT-3.5 and ChatGPT-4 based on the open-access status or the publication date. Both GPT-3.5 and GPT-4 models were constructed using publicly available databases and the knowledge cutoffs set in early 2021 [16,26]. Therefore, we hypothesized that open access case reports could produce better diagnostic results than non–open access ones. Additionally, we postulated that the case reports published in the years prior to 2021 could produce better diagnostic results than the ones published in 2022. The actual results were partly attributed to the limited sample size resulting from the subdivision into exploratory analysis.

### Potential Implications for Clinical Practice and Medical Education

The integration of generative AI like ChatGPT into clinical settings could enhance patient care and streamline physician workflows. Given its pretraining accuracy of over 80%, physicians could receive immediate support in challenging cases, thereby minimizing diagnostic errors and enhancing patient outcomes. Furthermore, these AI systems could grant health care professionals more time for the demanding facets of patient care, allowing them to focus on more demanding aspects of patient care and potentially thereby improving health care efficiency. In an educational context, ChatGPT could be pivotal in shaping future physicians, especially in clinical reasoning and medical knowledge acquisition [27]. Engaging with generative AIs can expose medical learners to an array of diagnoses, preparing them for complex clinical situations.

### Limitations

This study has several limitations. First, the study materials were obtained solely from complex case reports published by a single GIM department at a single center. Although these case reports provided insight into challenging diagnostic scenarios, they may not capture the full spectrum of patient presentations, even within the GIM department, as they were not randomly sampled but rather selected for their complexity, unusualness, or the challenges they posed for diagnosis. Therefore, our findings have limited external validity, as they may not be generalizable to other settings. Their performance might differ in simpler or more typical clinical presentations. Second, we acknowledge the possible bias in the differential diagnosis lists. They were created by experienced GIM physicians, implying that the results might not be applicable to lists created by physicians of different specialties or with various levels of training. It would be beneficial if future studies incorporated a wider array of participants. Third, there is a limitation associated with the accessibility and recency of our study. Specifically, 75% (39/52) of the case studies were published as open access, and approximately half of the case studies were published prior to 2021. Although we did not observe statistically significant differences regarding open access and publication timing, there were some possibilities for ChatGPT-3.5, ChatGPT-4, and physicians who created differential diagnosis lists to learn these case materials directly or indirectly. The final limitation pertains to possible time lag when generating differential diagnosis lists between ChatGPT-3.5 and ChatGPT-4. In light of these limitations, future research should assess the diagnostic accuracy of ChatGPT models by using properly tuned case materials that the model has not been trained on.

### Comparison With Prior Work

Our previous study [22] showed that the diagnostic accuracy of ChatGPT-3.5 was lower than that of physicians (25/30, 83% vs 59/60, 98%, respectively). In contrast, the findings of this study revealed that the rates of correct diagnoses within the top 10 (43/52, 83% vs 39/52, 75%, respectively) and top 5 (42/52, 81% vs 35/52, 67%, respectively) differential diagnosis lists, as well as the top diagnosis (31/52, 60% vs 26/52, 50%, respectively) generated by ChatGPT-4 were comparable to those by physicians. These results suggest the evolving performance of AI chatbots across different ChatGPT versions. Compared with those in the prior study [22], the rates of correct diagnoses within the top 10 (38/52, 73% vs 28/30, 93%, respectively) and top 5 (34/52, 65% vs 25/30, 83%, respectively) differential diagnosis lists and top diagnosis (22/52, 42% vs 16/30, 53%, respectively) generated by ChatGPT-3 (or 3.5) were lower in this study. This discrepancy was largely attributed to this study’s emphasis on complex clinical case vignettes sourced from case reports within the GIM department, while the prior research focused on more common clinical presentations. Moreover, ChatGPT-4 provided better results in its differential diagnosis lists (43/52, 83% vs 45/70, 64%, respectively) and as its top diagnosis (31/52, 60% vs 27/70, 39%, respectively) compared with those reported in another study for New England Journal of Medicine clinicopathologic conferences [21]. These variations can be partly ascribed to differences in the study designs, including case vignettes and systems.

Compared with a previous review on symptom checkers [5], the rate of correct diagnoses within the top 10 differential diagnoses generated by ChatGPT-4 was higher (43/52, 83% vs 60.9%-76.9%, respectively) in this study. Compared with a previous review on the differential diagnosis generator [6], the rate of correct diagnoses within the top 10 differential diagnoses generated by ChatGPT-4 was higher (43/52, 83% vs 63%-77%, respectively) in this study. This discrepancy is partly due to differences in study designs, case materials, and algorithms. In the future, direct comparisons between ChatGPT and other CDS systems are required.

### Conclusions

This study demonstrates the potential diagnostic accuracy of the differential diagnosis lists generated by ChatGPT-3.5 and ChatGPT-4 by using complex clinical vignettes from case reports published by the GIM department. Notably, the rate of correct diagnoses within the top 10 and top 5 differential diagnosis lists generated by ChatGPT-4 exceeds 80%. Although these results stem from a limited data set of case reports from a single department, they indicate the potential utility of ChatGPT-4 as a supplementary tool for physicians, particularly for those affiliated with the GIM department. Future research should assess the diagnostic accuracy of ChatGPT models by using properly tuned case materials that the model has not been trained on. Additionally, future investigations should evaluate the literacy level of AIs and their alignment with relevant medical text. Such efforts will ensure a comprehensive insight into the AI’s possible roles in enhancing clinical decision-making processes. Moreover, as AI systems become more prevalent, their influence is expected to ripple across various facets of health care. Generative AIs have the potential to reshape patient-physician dynamics, fostering more informed interactions. They can also play a pivotal role in democratizing medical knowledge. This could lead to heightened health care accessibility, allowing even those in remote or underserved regions to glean expert medical advice. Given these profound implications, it becomes imperative to investigate the ramifications of AI integration into health care.

## Appendix

Multimedia Appendix 1 Case reports included in this study.

Multimedia Appendix 2 Final diagnosis and the differential diagnosis lists generated by ChatGPT and those created by physicians.

## Abbreviations

AI: artificial intelligence
CDS: clinical decision support
GIM: general internal medicine
GPT: generative pretrained transformer
GPT-3.5: third-generation generative pretrained transformer
GPT-4: fourth-generation generative pretrained transformer
