# Supplementary material for: ChatGPT-Generated Differential Diagnosis Lists for Complex Case–Derived Clinical Vignettes: Diagnostic Accuracy Evaluation
Source: JMIR Med Inform. 2023 Oct 9;11:e48808. doi: 10.2196/48808 (PMC10594139; doi:10.2196/48808)
Supplement: Multimedia Appendix 1 [file medinform_v11i1e48808_app1.pdf]

Table S1. Case reports included in this study.

Number	The title of case reports	URL	Open-access	Published year
1	Diagnosing Bilateral Tarsal Tunnel Syndrome	https://www.amjmed.com/article/S0002-9343(17)30579-X/pdf	1	2017
2	Non-episodic angioedema associated with eosinophilia	https://casereports.bmj.com/content/15/6/e250037.long	1	2022
3	Hepatic portal venous gas after diving	https://www.ncbi.nlm.nih.gov/pmc/articles/PMC5780594/	1	2018
4	18F-Fluorodeoxyglucose positron emission tomography computed	https://www.ncbi.nlm.nih.gov/pmc/	1	2021

	tomography detection of single organ vasculitis of the breast: A case report	articles/PMC9281965/		
5	A Case of Multiple Ring-Shaped Aphthae Without Pseudomembranous Colitis	https://www.amjmedsci.org/article/S0002-9629(21)00284-6/fulltext	0	2022
6	Difficult differentiation of a somatic symptom disorder from anterior cutaneous nerve entrapment syndrome (ACNES): a case report	https://bmcpsychiatry.biomedcentral.com/counter/pdf/10.1186/s12888-019-2390-2.pdf	1	2019
7	Acute upper airway obstruction by a goitre due to Hashimoto's thyroiditis	https://casereports.bmj.com/content/14/9/e245198.long	1	2021
8	Two Cases of Acute Urinary Retention Associated With Acute Sarcopenia in	https://assets.cureus.com/uploads	1	2022

	Older Women	/case_report/pdf/114330/20221022-7062-1yxvt9r.pdf		
9	An Arterial Perihepatic Enhancement Caused by Spontaneous Bladder Rupture	https://www.goldjournal.net/article/S0090-4295(16)30912-8/fulltext	0	2017
10	Calcinosis cutis causing cutaneous ulceration and secondary bacterial infection in a patient with antinuclear matrix protein 2 antibody-positive dermatomyositis	https://onlinelibrary.wiley.com/doi/pdfdirect/10.1002/ams2.810?download=true	1	2022
11	The remains of the D	https://onlinelibrary.wiley.com/doi/pdfdirect/10.1002/jgf2.563?downlo	1	2022

		ad=true		
12	Hyperintense central pontine lesion in intravascular large B-cell lymphoma	https://casereports.bmj.com/content/15/9/e250945.long	1	2022
13	Characteristic speech qualities associated with hypothyroidism	https://casereports.bmj.com/content/15/3/e244173.long	1	2022
14	Gluten-Related Disorder and Lactose Intolerance Concomitant With Avoidant/Restrictive Food Intake Disorder	https://www.psychiatrist.com/pcc/eating/gluten-disorder-and-lactose-intolerance-concomitant-with-avoidantrestrictiv	1	2020

		e-food-intake-disorder/		
15	Delayed diagnosis of a piriformis abscess due to hyposkillia and communication error	https://casereports.bmj.com/content/14/8/e242088.long	1	2021
16	Pazopanib-associated interstitial lung disease in a patient with renal cell carcinoma	https://www.ncbi.nlm.nih.gov/pmc/articles/PMC7348321/	1	2020
17	Case of bilateral Bell's palsy	https://casereports.bmj.com/content/15/6/e250364.long	1	2022
18	Gastric Anisakiasis	https://www.amjmedsci.org/article/S0002-9629(20)30152-	0	2020

		X/fulltext		
19	Unilateral spatial neglect	https://www.ncbi.nlm.nih.gov/pmc/articles/PMC7684669/	1	2020
20	Recurrent Hematoma in the Finger and Leg: Achenbach Syndrome	https://www.amjmed.com/article/S0002-9343(21)00438-1/fulltext	0	2021
21	Popliteal Ecchymosis in Ruptured Baker's Cyst	https://www.amjmed.com/article/S0002-9343(20)30926-8/fulltext	0	2021
22	Asymptomatic syndrome of inappropriate secretion of antidiuretic hormone (SIADH) following duloxetine treatment for pain	https://onlinelibrary.wiley.com/doi/pdfdirect/10.1002	1	2022

	with depression: Two case reports	/npr2.12279?download=true		
23	SAPHO syndrome	https://www.amjmedsci.org/article/S0002-9629(19)30287-3/fulltext	0	2020
24	Behind the Leg: Cryptococcal Meningitis, Not Cellulitis	https://www.amjmed.com/article/S0002-9343(19)30550-9/fulltext	0	2020
25	Vertebral Artery Dissection after Exposure to Levofloxacin: A Report of Two Cases	https://www.jstage.jst.go.jp/article/internalmedicine/60/17/60_6736-20/_pdf	1	2021
26	SAPHO syndrome	https://pubmed.n	1	2019

		cbi.nlm.nih.gov/31796447/		
27	The systemic immune response due to cholesterol crystal embolization syndrome: a case report	https://bmcnephrol.biomedcentral.com/counter/pdf/10.1186/s12882-022-02939-9.pdf	1	2022
28	Diagnostic Difficulties in Treating a Typical Case of a Patient Being a Doctor	https://www.amjmed.com/article/S0002-9343(20)30551-9/fulltext	0	2021
29	Secretion from umbilicus	https://www.ncbi.nlm.nih.gov/pmc/articles/PMC6700552/	1	2019
30	Lorazepam as a Cause of Drug-Induced Liver Injury	https://www.karger.com/Article/Pd	1	2018

		f/492209		
31	Erythema nodosum-like lesion on heel in a patient assumed Behçet's disease	https://www.ncbi.nlm.nih.gov/pmc/articles/PMC6887397/	1	2019
32	Severe leptospirosis in a patient with positive serological test for spotted fever rickettsiosis	https://www.ncbi.nlm.nih.gov/pmc/articles/PMC6340506/	1	2019
33	Clozapine-associated severe eosinophilia following lithium rebound neutropenia: A case report	https://onlinelibrary.wiley.com/doi/pdfdirect/10.1002/npr2.12143?download=true	1	2020
34	Brachiocephalic Vein Thrombosis	https://www.amjmedsci.org/article/S0002-9629(19)30430-	0	2020

		6/fulltext		
35	The challenge of differentiating tuberculous meningitis from bacterial meningitis	https://onlinelibrary.wiley.com/doi/pdfdirect/10.1002/rcr2.910?download=true	1	2022
36	Missed Opportunities for Diagnosing Vertebral Osteomyelitis Caused by Influential Cognitive Biases	https://amjcaserep.com/abstract/full/idArt/936058	1	2022
37	Skin and soft tissue infections and bacteremia caused by Vibrio cincinnatiensis	https://www.ncbi.nlm.nih.gov/pmc/articles/PMC9278064/	1	2022
38	Left pleural effusion caused by pancreaticopleural fistula with a pancreatic pseudocyst	https://www.ncbi.nlm.nih.gov/pmc/articles/PMC5015151/	1	2016
39	Cerebral Venous Thrombosis	https://www.amj	0	2019

		medsci.org/article/S0002-9629(19)30099-0/fulltext		
40	Acute cytomegalovirus infection in a 61-year-old woman	https://www.cmaj.ca/content/cmaj/194/32/E1109.full.pdf	1	2022
41	Fitz-Hugh-Curtis syndrome without salpingitis: Should contrast-enhanced computed tomography be a routine diagnostic procedure?	https://onlinelibrary.wiley.com/doi/pdfdirect/10.1002/ccr3.5211?download=true	1	2021
42	Aortic intramural haematoma associated with pulmonary artery periadventitial haematoma	https://www.ncbi.nlm.nih.gov/pmc/articles/PMC5965761/	1	2018
43	Is a Gallbladder Coming Back? A Seroma	https://www.amj	0	2021

	in the Gallbladder Fossa	med.com/article/ S0002- 9343(20)30927- X/fulltext		
44	Hypertrophic pachymeningitis in the context of the treatment of polymyalgia rheumatica	https://casereports.bmj.com/content/15/6/e250450.long	1	2022
45	Achenbach syndrome	https://www.cmaj.ca/content/cmaj/191/21/E584.full.pdf	1	2019
46	Delayed Diagnosis of Pulmonary Embolism	https://www.amjmed.com/article/S0002-9343(21)00810-X/fulltext	0	2022
47	Atypical presentation of colorectal	https://jmedicalca	1	2021

	carcinoma with sole multiple osteolytic bone metastases: a case report	sereports.biomedcentral.com/counter/pdf/10.1186/s13256-021-02795-5.pdf		
48	A Case of Ramsay Hunt Syndrome That Began with Vestibular Symptoms: A Great Mimicker	https://www.amjmed.com/article/S0002-9343(20)30936-0/fulltext	0	2021
49	Three Cases of Food Poisoning Due to Paralepistopsis acromelalga Diagnosed from an Outbreak of Erythromelalgia	https://www.jstage.jst.go.jp/article/internalmedicine/60/10/60_4650-20/_pdf	1	2021
50	Microangiopathic haemolytic anaemia with thrombocytopenia induced by vitamin B12 deficiency long term after	https://www.ncbi.nlm.nih.gov/pmc/articles/PMC615	1	2018

	gastrectomy	0191/		
51	Osmotic demyelination syndrome due to hyperosmolar hyperglycemia	https://www.ccjm.org/content/ccjom/85/7/511.full.pdf	1	2018
52	Intramural haematoma of the oesophagus	https://www.ncbi.nlm.nih.gov/pmc/articles/PMC6626467/	1	2019

Notes: the number was same as the Table S2 of Multimedia Appendix 2.
