# Supplementary material for: ChatGPT-Generated Differential Diagnosis Lists for Complex Case–Derived Clinical Vignettes: Diagnostic Accuracy Evaluation
Source: JMIR Med Inform. 2023 Oct 9;11:e48808. doi: 10.2196/48808 (PMC10594139; doi:10.2196/48808)
Supplement: Multimedia Appendix 2 [file medinform_v11i1e48808_app2.pdf]

Table S2. Final diagnosis and the differential-diagnosis lists generated by Chat Generative Pre-Trained Transformers and those created by physicians.

Number	Final diagnosis	Differential-diagnosis list		
		ChatGPT-3	ChatGPT-4	Physicians
1	Tarsal tunnel syndrome	1. Peripheral neuropathy<br>2. Tarsal tunnel syndrome<br>3. Lumbar spinal stenosis<br>4. Diabete<br>5. Alcohol abuse<br>6. Vitamin B12 deficiency<br>7. Hypothyroidism<br>8. Chronic kidney disease	1. Peripheral neuropathy<br>2. Tarsal tunnel syndrome<br>3. Lumbar radiculopathy<br>4. Small fiber neuropathy<br>5. Chronic inflammatory demyelinating polyneuropathy (CIDP)	1. Sarcoidosis<br>2. Chronic inflammatory demyelinating polyradiculoneuropathy<br>3. Diabetic neuropathy<br>4. Vitamin B12 deficiency<br>5. Alcoholic neuropathy<br>6. Paraneoplastic

		9. Multiple sclerosis<br>10. Charcot-Marie-Tooth disease	6. Complex regional pain syndrome (CRPS)<br>7. Peripheral artery disease (PAD)<br>8. Charcot-Marie-Tooth disease<br>9. Polyneuropathy<br>10. Leprosy	syndrome<br>7. Anti-myelin oligodendrocyte glycoprotein antibody-associated disease<br>8. ANCA-related vasculitis<br>9. POEMS syndrome<br>10. Restless legs syndrome
2	Non-episodic angioedema associated with eosinophilia	1. Eosinophilic fasciitis<br>2. Acute interstitial nephritis<br>3. Hypereosinophili	1. Eosinophilic cellulitis (Wells syndrome)<br>2. Idiopathic eosinophilic	1. hypereosinophilic syndrome<br>2. idiopathic peripheral edema

c syndrome<br>4. Churg-Strauss syndrome<br>5. Idiopathic hypereosinophilic syndrome<br>6. Lymphoma<br>7. Systemic lupus erythematosus<br>8. Sarcoidosis<br>9. Infectious mononucleosis<br>10. Allergic reactions	oedema<br>3. Hypereosinophilic syndrome<br>4. Eosinophilic granulomatosis with polyangiitis (Churg-Strauss syndrome)<br>5. Drug-induced eosinophilia<br>6. Eosinophilic fasciitis (Shulman syndrome)<br>7. Kimura disease	3. idiopathic angioedema<br>4. cholinergic urticaria<br>5. eosinophilic granulomatosis with polyangiitis：EGPA<br>6. parasitic infection<br>7. chronic eosinophilic leukemia<br>8. Drug(include supplement, herabal organs)

			8. Eosinophilic gastroenteritis<br>9. Parasitic infections<br>10. Systemic lupus erythematosus (SLE)	induced hypereosinphilic edema<br>9. tuberculosis<br>10. viral infection
3	Decompression sickness	1. Arterial gas embolism<br>2. Decompression sickness<br>3. Carbon monoxide poisoning<br>4. Nitrogen narcosis<br>5. Hypothermia	1. Decompression sickness (DCS)<br>2. Gas embolism<br>3. Acute coronary syndrome (ACS)<br>4. Gastroenteritis<br>5. Acute	1. air embolisation<br>2. decompression sickness<br>3. non-occlusive mesenteric ischemia （NOMI）<br>4. diabetic ketoacidosis

		6. Acute pancreatitis<br>7. Mesenteric ischemia<br>8. Gastroenteritis<br>9. Gastric ulcer<br>10. Acute cholecystitis	pancreatitis<br>6. Peptic ulcer disease<br>7. Diabetic ketoacidosis (DKA)<br>8. Acute cholecystitis<br>9. Mesenteric ischemia<br>10. Gastritis	5. bacteremia<br>6. bacterial translocation<br>7. Acute coronary syndrome<br>8. cholelithiasis<br>9. cholangitis<br>10. cholesterol embolisation
4	Polyarteritis nodosa	1. Infection<br>2. Cancer<br>3. Vasculitis<br>4. Connective tissue disease<br>5. Granulomatous disease	1. Infection<br>2. Temporal arteritis<br>3. Polymyalgia rheumatica<br>4. Vasculitis<br>5. Malignancy	1. Mondor disease<br>2. ANCA negative Vascuritis (MPA)<br>3. ANCA

6. Malignant lymphoma<br>7. Thromboembolic disease<br>8. Drug-induced fever<br>9. Heat-related illness<br>10. Unknown fever	6. Autoimmune disease<br>7. Subacute bacterial endocarditis<br>8. Drug-induced fever<br>9. Lymphoma<br>10. Inflammatory breast cancer	negative Vascuritis (GPA)<br>4. Infectious endcardiitis<br>5. Cryoglobulinemia(due to Multiple Myeloma)<br>6. Cryoglobulinemia(due to hepatitis C)<br>7. Intravascular lymphoma<br>8. Bechet disease<br>9. Paraneoplastic syndrome

				10. Breast cancer
5	Clostridioides difficile infection	1. Clostridioides difficile infection<br>2. Inflammatory bowel disease<br>3. Diverticulitis<br>4. Irritable bowel syndrome<br>5. Colitis<br>6. Infectious diarrhea<br>7. Intestinal obstruction<br>8. Celiac disease<br>9. Colon cancer<br>10. Intestinal ischemia	1. Clostridioides difficile infection (CDI)<br>2. Inflammatory Bowel Disease (IBD)<br>3. Microscopic colitis<br>4. Ischemic colitis<br>5. Diverticulitis<br>6. Irritable Bowel Syndrome (IBS)<br>7. Infectious colitis<br>8. Food allergy or	1. Campylobacter enteritis<br>2. Cytomegalovirus enterocolitis<br>3. Clostridioides difficile infection<br>4. Acute HIV infection<br>5. Nonsteroidal anti-inflammatory drug-induced colitis<br>6. Intestinal Behçet's Disease

			intolerance<br>9. Gastroenteritis (viral or bacterial)<br>10. Celiac disease	7. Intestinal lupus<br>8. Crohn's Disease
6	Anterior cutaneous nerve entrapment syndrome	1. Adrenal insufficiency<br>2. Depression<br>3. Chronic fatigue syndrome<br>4. Irritable bowel syndrome (IBS)<br>5. Fibromyalgia<br>6. Anxiety disorder<br>7. Gastritis or peptic ulcer disease<br>8. Endometriosis	1. Adrenal insufficiency<br>2. Irritable bowel syndrome (IBS)<br>3. Functional dyspepsia<br>4. Gastroesophageal reflux disease (GERD)<br>5. Chronic fatigue	1. Anterior cutaneous nerve entrapment syndrome<br>2. Corticosteroid-binding globulin deficiency<br>3. Inguinal hernia<br>4. Congenital heart disease

		9. Polycystic ovary syndrome (PCOS)<br>10. Inflammatory bowel disease (IBD)	syndrome (CFS)<br>6. Anxiety disorder<br>7. Somatoform disorder<br>8. Celiac disease<br>9. Endometriosis<br>10. Pelvic inflammatory disease (PID)	
7	Upper airway obstruction by the diffusely enlarged thyroid	1. Acute respiratory distress syndrome (ARDS)<br>2. Pulmonary embolism	1. Thyroid-related respiratory distress<br>2. Chronic obstructive pulmonary	1. Negative pressure pulmonary edema<br>2. Hashimoto's thyroiditis<br>3. Beriberi heart

3. Hypothyroidism
4. Thyroid storm
5. Acute exacerbation of chronic obstructive pulmonary disease (COPD)
6. Pneumonia
7. Acute heart failure
8. Bronchial asthma
9. Atrial fibrillation
10. Pulmonary edema

disease (COPD)

3. Congestive heart failure
4. Pneumonia
5. Pulmonary embolism
6. Asthma
7. Respiratory acidosis
8. Acute respiratory distress syndrome (ARDS)
9. Anaphylaxis
10. Aspiration pneumonitis

8	Acute urinary retention associated with acute sarcopenia	1. Malignant syndrome<br>2. Parkinson's disease<br>3. Poor medication adherence<br>4. Urinary tract infection<br>5. Dehydration<br>6. Malnutrition<br>7. Sarcopenia<br>8. Impaired mobility<br>9. Frailty<br>10. Cognitive impairment	1. Neuroleptic Malignant Syndrome (NMS)<br>2. Urinary Tract Infection (UTI)<br>3. Sarcopenia<br>4. Parkinson's Disease Progression<br>5. Medication Side Effects<br>6. Dehydration<br>7. Constipation<br>8. Orthostatic Hypotension<br>9. Depression or Anxiety	1. wearing off phenomenon of Parkinson's Disease<br>2. hypoactive delirium<br>3. Disuse syndrome<br>4. Drug-induced sarcopenia<br>5. Exacerbations of Parkinson's disease<br>6. Drug-induced myopathy<br>7. hospitalization induced sarcopenia

			10. Malnutrition	8. dimentia<br>9. aspiration pneumonia<br>10. Acute kideny injury
9	Spontaneous bladder rupture	1. Bladder perforation<br>2. Peritonitis<br>3. Urinary tract infection<br>4. Bowel obstruction<br>5. Appendicitis<br>6. Colon cancer recurrence<br>7. Diverticulitis<br>8. Pelvic inflammatory	1. Peritonitis<br>2. Bowel obstruction<br>3. Bowel perforation<br>4. Diverticulitis<br>5. Urinary tract infection (UTI)<br>6. Radiation enteritis<br>7. Adhesions<br>8. Ischemic colitis	1. panperitonitis(due to bladder rupture)(due to post-radiation scar)<br>2. panperitonitis(due to bladder rupture)(due to bladder cancer)<br>3. panperitonitis(due to bladder rupture)(due to

	disease<br>9. Gastrointestinal perforation<br>10. Abdominal trauma	9. Cholecystitis<br>10. Appendicitis	rectal cancer metasis)<br>4. panperitonitis(due to bladder rupture)(due to ovarian cancer metasis)<br>5. panperitonitis(due to bladder rupture)(due to ovarian cancer metasis)<br>6. panperitonitis(due to bladder rupture)(due to endometrial cancer metasis)

				7. panperitonitis(due to bladder rupture)(due to emphysematous cystitis)<br>8. panperitonitis(due to bladder rupture)(due to injury)<br>9. panperitonitis(due to bladder rupture)(urethral obstruction)<br>10. pelvic inflammatory disease
10	Ulcerated calcinosis cutis	1. Calciphylaxis<br>2. Osteomyelitis	1. Calciphylaxis<br>2. Tophaceous	1. Systemic sclerosis

3. Septic arthritis
4. Cellulitis
5. Pyomyositis
6. Necrotizing fasciitis
7. Gout
8. Rheumatoid arthritis
9. Systemic lupus erythematosus (SLE)
10. Raynaud’s disease

gout
3. Secondary infection
4. Osteomyelitis
5. Rheumatoid arthritis
6. Scleroderma
7. CREST syndrome
8. Pyogenic granuloma
9. Vasculitis
10. Chronic regional pain syndrome (CRPS)

2. Mixed connective tissue disease
3. Microscopic polyangiitis
4. Infectious endcardiitis
5. Cutaneous Mycobacterial Infections
6. Peripheral artery disease
7. Bechet disease
8. Intravascular lymphoma
9. Skin Cancer
10. Injury/burn

11	Hypercalcemia caused by new-type calcium-alkali syndrome	1. Gastrointestinal (GI) disorders<br>2. Anemia<br>3. Dehydration<br>4. Hypocalcemia<br>5. Renal failure<br>6. Hypercalcemia<br>7. Stroke<br>8. Dementia<br>9. Medication side effects<br>10. Osteoporosis	1. Dehydration<br>2. Acute kidney injury<br>3. Hypercalcemia<br>4. Anemia<br>5. Infection<br>6. Medication side effects<br>7. Malnutrition<br>8. Worsening vascular dementia<br>9. Hypomagnesemia<br>10. Congestive heart failure	1. Drug-induced hypercalcemia<br>2. Drug-induced somnolence<br>3. Hypercalcemia with nultiple mueloma<br>4. Nonconvulsive status epilepticus<br>5. Meningoencephalitis<br>6. Septic encephalopathy<br>7. Hypercalcemia with PTH-rp<br>8. Thrombotic

				microangiopathy<br>9. Wernicke encephalopathy<br>10. Hashimoto's encephalopathy
12	Intravascular large B-cell lymphoma	1. Hemophagocytic lymphohistiocytosis (HLH)<br>2. Central nervous system (CNS) lymphoma<br>3. B-cell lymphoma<br>4. Multiple	1. B-cell lymphoma<br>2. Autoimmune lymphoproliferative syndrome (ALPS)<br>3. Hemophagocytic lymphohistiocy	1. Intravascular large B cell lymphoma

myeloma<br>5. Systemic lupus erythematosus (SLE)<br>6. Wegener’s granulomatosis<br>7. Hepatitis B or C<br>8. Malaria<br>9. Dengue fever<br>10. Leptospirosis	tosis (HLH)<br>4. Systemic lupus erythematosus (SLE)<br>5. Vasculitis<br>6. Infectious mononucleosis<br>7. Chronic lymphocytic leukemia (CLL)<br>8. Myelodysplastic syndrome (MDS)<br>9. Paraneoplastic syndrome:

			10. Central nervous system (CNS) infection or inflammation	
13	Hashimoto thyroidisits	1. Graves’ disease<br>2. Myasthenia gravis<br>3. Hypothyroidism<br>4. Polymyositis<br>5. Inclusion body myositis<br>6. Hyperlipidemia<br>7. Cushing’s syndrome<br>8. Fibromyalgia<br>9. Multiple	1. Hypothyroidism<br>2. Myasthenia gravis<br>3. Polymyositis<br>4. Dermatomyositis<br>5. Inclusion body myositis<br>6. Muscular dystrophy<br>7. Lambert-Eaton syndrome	1. Myathenia gravis with Hashimoto's thyroiditis<br>2. Amyotrophic lateral sclerosis with Hashimoto's thyroiditis<br>3. Giant cell artelitis<br>4. Takayasu's arteritis

		sclerosis<br>10. Amyotrophic lateral sclerosis (ALS)	8. Multiple sclerosis<br>9. Guillain-Barré syndrome<br>10. Amyotrophic lateral sclerosis (ALS)	
14	Gluten-related disorder	1. Crohn’s Disease<br>2. Celiac Disease<br>3. Pancreatic Insufficiency<br>4. Hyperthyroidism<br>5. Tuberculosis<br>6. Addison’s Disease	1. Celiac disease<br>2. Inflammatory bowel disease (IBD)<br>3. Irritable bowel syndrome (IBS)<br>4. Chronic pancreatitis<br>5. Food allergies	1. Celiac disease<br>2. Wheat allergy<br>3. Hereditary angioedema<br>4. Ulcerative colitis<br>5. Crohn's disease<br>6. HIV<br>7. Lupus enteritis

		7. Anorexia Nervosa<br>8. Bulimia Nervosa<br>9. Gastroparesis<br>10. Malignancy	or intolerances<br>6. Small intestinal bacterial overgrowth (SIBO)<br>7. Gastrointestinal infections<br>8. Eating disorders<br>9. Malabsorption syndromes<br>10. Addison's disease	8. Intestinal tuberculosis<br>9. Depression<br>10. Anorexia nervosa
15	Piriformis abscess	1. Pyelonephritis<br>2. Prostatitis<br>3. Osteomyelitis<br>4. Septicemia	1. Pyelonephritis<br>2. Complicated urinary tract infection	1. Piriformis muscle abcess(due to infectious

		5. Cellulitis<br>6. Epidural abscess<br>7. Discitis<br>8. Spondylodiscitis<br>9. Staphylococcus aureus infection<br>10. Piriformis syndrome	3. Prostatits<br>4. Septic arthritis<br>5. Epidural abscess<br>6. Vertebral osteomyelitis<br>7. Psoas abscess<br>8. Discitis<br>9. Diverticulitis<br>10. Pelvic inflammatory disease	endcardiitis)<br>2. Piriformis muscle abcess(due to<br>3. direct infiltration of renal abcess)<br>4. Piriformis muscle abcess(due to<br>5. direct infiltration of pressure ulcer infection)
16	Drug-induced interstitial lung disease	1. Pulmonary embolism<br>2. Pneumonia<br>3. Metastatic	1. Pneumonia<br>2. COVID-19<br>3. Pulmonary metastasis	1. irAe: Immune-related Adverse Events

lung cancer<br>4. Gastrointestinal bleeding<br>5. Hypoalbuminemia<br>6. Thyroid cancer<br>7. Chronic obstructive pulmonary disease (COPD)<br>8. Pulmonary fibrosis<br>9. Bronchitis<br>10. COVID-19	4. Drug-induced lung injury<br>5. Pulmonary embolism<br>6. Radiation pneumonitis<br>7. Bronchitis<br>8. Diverticular bleeding<br>9. Lower gastrointestinal bleeding<br>10. Thyroid disorder	2. Drug-induced lung injury<br>3. Radiation-induced lung injury<br>4. cancer related lymphangitis<br>5. organizing pneumonia<br>6. Pneumocystis Jirovecii Pneumonia<br>7. Invasive fungal infection<br>8. atypical pneumonia<br>9. community aquired

				pneumonia<br>10. tuberculosis
17	Bell’s palsy	1. Bell’s palsy<br>2. Trigeminal neuralgia<br>3. Stroke<br>4. Temporal arteritis<br>5. Migraine<br>6. Cluster headache<br>7. Sinusitis<br>8. Myasthenia gravis<br>9. Lyme disease<br>10. Ramsay Hunt syndrome	1. Bell's Palsy<br>2. Guillain-Barré Syndrome<br>3. Lyme Disease<br>4. Ramsay Hunt Syndrome<br>5. Brainstem Stroke<br>6. Diabetic Neuropathy<br>7. Sarcoidosis<br>8. Multiple Sclerosis<br>9. Myasthenia Gravis<br>10. Vasculitis	1. stroke of brain stem<br>2. bilateral facial nerve paralysis<br>3. neurosyphilis<br>4. Bell's palsy<br>5. giant cell arthritis<br>6. encephalopath y<br>7. drug-induced neuropahy<br>8. parkinson's disease

18	Gastric anisakis	1. Gastric Anisakiasis<br>2. Acute Gastritis<br>3. Peptic Ulcer Disease<br>4. Acute Pancreatitis<br>5. Gastroenteritis<br>6. Cholecystitis<br>7. Acute Appendicitis<br>8. Biliary Colic<br>9. Intestinal Obstruction<br>10. Acute Myocardial Infarction	1. Anisakiasis<br>2. Gastroenteritis<br>3. Acute gastritis<br>4. Peptic ulcer disease<br>5. Acute pancreatitis<br>6. Acute cholecystitis<br>7. Acute appendicitis<br>8. Peritonitis<br>9. Bowel obstruction<br>10. Ectopic pregnancy	1. Gastric anisakiasis<br>2. Acute gastric mucosal lesion<br>3. Gastric cancer
19	Acute	1. Stroke	1. Ischemic	1. Brain

	ischaemic stroke	2. Hemispatial neglect<br>3. Traumatic brain injury<br>4. Brain tumor<br>5. Dementia<br>6. Encephalitis<br>7. Migraine<br>8. Parkinson’s disease<br>9. Multiple sclerosis<br>10. Alzheimer’s disease	Stroke<br>2. Transient Ischemic Attack (TIA)<br>3. Hemianopia<br>4. Hemispatial neglect<br>5. Seizure<br>6. Migraine with aura<br>7. Brain tumor<br>8. Encephalitis<br>9. Dementia<br>10. Functional neurological disorder	Infarction(right inferior parietal lobule)<br>2. Cerebral sinus thrombosis<br>3. Hypertensive encephalopathy<br>4. Moyamoya disease<br>5. cerebral arteriovenous malformation
20	Achenbach Syndrome	1. Raynaud’s disease	1. Idiopathic spontaneous	1. Achenbach syndrome

	subcutaneous hematomas	
2. Thromboangiitis obliterans (Buerger’s disease)	2. Hereditary angioedema	2. Scurvy
3. Vasculitis	3. Acute idiopathic blue finger	3. von Willebrand disease
4. Hypereosinophilic syndrome	4. Raynaud's phenomenon	
5. Scleroderma	5. Erythromelalgia	
6. Cryoglobulinemia	6. Paroxysmal hand hematoma	
7. Lupus	7. Vasculitis	
8. Acute compartment syndrome	8. Pseudoxanthoma elasticum	
9. Deep vein thrombosis	9. Ehlers-Danlos	
10. Hemophilia		

			syndrome<br>10. Heme-related disorders	
21	Ruptured Baker’s cyst	1. Baker’s cyst<br>2. Popliteal artery aneurysm<br>3. Popliteal vein aneurysm<br>4. Popliteal artery entrapment syndrome<br>5. Thrombophlebitis<br>6. Torn meniscus<br>7. Osteoarthritis<br>8. Rheumatoid arthritis<br>9. Gout	1. Baker's cyst (Popliteal cyst)<br>2. Calf muscle tear or strain<br>3. Hematoma<br>4. Bursitis<br>5. Tendinitis<br>6. Osteoarthritis<br>7. Meniscal tear<br>8. Knee ligament injury<br>9. Gastrocnemius muscle rupture<br>10. Nerve	1. Rupture of Baker cyst<br>2. Septic arthritis of the knee<br>3. Osteoarthritis of the knee<br>4. Elderly-onset rheumatoid arthritis

		10. Septic arthritis	entrapment	
22	Syndrome of inappropriate secretion of antidiuretic hormone	1. Syndrome of inappropriate antidiuretic hormone secretion (SIADH)<br>2. Drug-induced hyponatremia<br>3. Adrenal insufficiency<br>4. Hypothyroidism<br>5. Multiple myeloma<br>6. Chronic kidney disease<br>7. Congestive	1. Syndrome of Inappropriate Antidiuretic Hormone Secretion (SIADH)<br>2. Medication-induced hyponatremia<br>3. Chronic kidney disease<br>4. Hypothyroidism<br>5. Adrenal insufficiency<br>6. Diabetic neuropathy	1. Duloxetine induced SIADH<br>2. NaCl intake deficiency<br>3. severe hypovolemia<br>4. Malignancy induced SIADH<br>5. liver cirrhosis<br>6. hyperglycemia<br>7. cerebral salt wasting syndrome<br>8. Corticosteroid-binding

		heart failure<br>8. Liver disease<br>9. Dehydration<br>10. Infection	7. Vitamin B12 deficiency<br>8. Peripheral neuropathy<br>9. Anxiety and depression<br>10. Drug interactions	globulin deficiency<br>9. renal tubular acidosis
23	SAPHO syndrome	1. Costochondriti s<br>2. Sterno-clavicular joint arthritis<br>3. Polymyalgia rheumatica<br>4. Giant cell arteritis<br>5. Rheumatoid	1. Sapho Syndrome<br>2. Osteoarthritis<br>3. Seronegative Spondyloarthr opathy<br>4. Costochondriti s<br>5. Tietze Syndrome	1. Tieze syndrome<br>2. Osteomyelitis of sternocostocla vicular joint<br>3. Sternoclavicul ar joint dislocation(du e to injory)

		arthritis<br>6. Ankylosing spondylitis<br>7. Sarcoidosis<br>8. Vasculitis<br>9. Fibromyalgia<br>10. Myocardial infarction	6. Infectious Arthritis<br>7. Sternoclavicular Septic Arthritis<br>8. Angina Pectoris<br>9. Myocardial Infarction<br>10. Inflammatory Myopathy	4. Rheumatoid arthritis<br>5. Palmoplantar pustulosis
24	Cryptococcal meningitis	1. Cryptococcal meningitis<br>2. Systemic fungal infection<br>3. Bacterial infection	1. Cryptococcal meningitis<br>2. Cellulitis<br>3. Immunosuppression-related infections<br>4. Cytomegalovir	1. Cryptococcal meningitis<br>2. HIV

		4. Osteomyelitis<br>5. Sepsis<br>6. Drug reaction<br>7. Endocarditis<br>8. Acute renal failure<br>9. Diabetes mellitus complications<br>10. Immunocompromised state	us (CMV) infection<br>5. Drug-induced fever<br>6. Urinary tract infection (UTI)<br>7. Bacterial meningitis<br>8. Endocarditis<br>9. Osteomyelitis<br>10. Deep vein thrombosis (DVT)	
25	Vertebral artery dissection	1. Ischemic Stroke<br>2. Vertebral Artery Dissection	1. Lateral medullary syndrome (Wallenberg syndrome)	1. Vertebrobasilar artery dissection (due to levofloxacin)

	3. Multiple Sclerosis<br>4. Brain Tumor<br>5. Guillain-Barre Syndrome<br>6. Lyme Disease<br>7. Syringomyelia<br>8. Cervical Disc Herniation<br>9. Arteriovenous Malformation (AVM)<br>10. Transient Ischemic Attack (TIA)	2. Vertebral artery dissection<br>3. Cerebellar infarction<br>4. Brainstem stroke<br>5. Intracranial hemorrhage:<br>6. Multiple sclerosis<br>7. Infection or inflammation of the central nervous system<br>8. Vasculitis<br>9. Cervical artery	2. Vertebrobasilar artery dissection (due to hypertension)<br>3. Ehlers-danlos syndrome<br>4. Syphilitic aortitis<br>5. Autosomal dominant polycystic kidney disease

			dissection<br>10. Complication from levofloxacin	
26	SAPHO syndrome	1. Chronic osteomyelitis<br>2. Sternocostoclavicular hyperostosis<br>3. SAPHO syndrome<br>4. Tietze syndrome<br>5. Fibromyalgia<br>6. Costochondritis<br>7. Coronary artery disease	1. SAPHO syndrome (Synovitis, Acne, Pustulosis, Hyperostosis, Osteitis)<br>2. Chronic osteomyelitis<br>3. Sternoclavicular joint septic arthritis<br>4. Inflammatory chest wall	1. SAPHO syndrome<br>2. ankylosing spondylitis<br>3. seronegative arthritis including psoriatic arthritis<br>4. Reactive arthritis (I.e. due to syphilis)<br>5. PMR<br>6. Diabetic

8. Angina
9. Myocardial infarction (heart attack)
10. Rheumatoid arthritis

syndrome (Tietze's syndrome or costochondritis)

5. Psoriatic arthritis
6. Ankylosing spondylitis
7. Atypical angina or coronary artery disease
8. Fibromyalgia
9. Osteoarthritis of the sternoclavicular joint

thoracic radiculopathy

7. Teitze syndrome
8. TAFRO syndrome
9. Malignant lymphoma

			10. Non-specific chest wall pain	
27	Cholesterol crystal embolization syndrome	1. Ventilator-associated pneumonia<br>2. Drug-induced erythema<br>3. Methicillin-resistant coagulase-negative staphylococcal infection<br>4. Acute tubulointerstitial nephritis<br>5. Eosinophilic vasculitis	1. Ventilator-associated pneumonia (VAP)<br>2. Methicillin-resistant coagulase-negative Staphylococcal infection<br>3. Drug-induced erythema<br>4. Acute kidney injury (AKI)<br>5. Drug-induced tubulointerstiti	1. Hypereosinophilic syndrome due to hypersensitivity to daptomycin/vancomycin<br>2. Hypereosinophilic syndrome due to hypersensitivity to stent graft<br>3. Eosinophilic granulomatosis with polyangiitis

6. Alcoholic hepatitis<br>7. Thoracic aneurysm rupture<br>8. Urinary tract obstruction<br>9. Hypertension<br>10. Proteinuria	al nephritis<br>6. Eosinophilic vasculitis<br>7. Urinary tract obstruction<br>8. Drug reaction with eosinophilia and systemic symptoms (DRESS) syndrome<br>9. Leukocytoclastic vasculitis<br>10. Systemic lupus erythematosus (SLE) or other	4. Infective endocarditis

			autoimmune diseases	
28	Acute appendicitis	1. Acute appendicitis<br>2. Urinary tract infection (UTI)<br>3. Gastroenteritis<br>4. Sushi-related food poisoning<br>5. Acute cystitis<br>6. Pyelonephritis<br>7. Pelvic inflammatory disease (PID)<br>8. Diverticulitis<br>9. Kidney stones<br>10. Inflammatory bowel disease	1. Acute appendicitis<br>2. Gastroenteritis<br>3. Food poisoning<br>4. Urinary tract infection (UTI)<br>5. Prostatitis<br>6. Diverticulitis<br>7. Pelvic inflammatory disease (PID)<br>8. Kidney stones<br>9. Irritable bowel syndrome (IBS)	1. Acute appenditis<br>2. Diverticulitis<br>3. Acute entelitis<br>4. Ersenia enterocolitis<br>5. Intestinal Behcet disease<br>6. intestinal tuberculosis<br>7. Crohn disease<br>8. ulcerative colitis<br>9. Fitting of a cuspid in the

		(IBD)	10. Inflammatory bowel disease (IBD)	appendix
29	Urachal remnant	1. Umbilical infection<br>2. Umbilical abscess<br>3. Omphalitis<br>4. Umbilical granuloma<br>5. Umbilical hernia<br>6. Diverticulitis<br>7. Appendicitis<br>8. Pelvic inflammatory disease (PID)<br>9. Gastroenteritis	1. Infected urachal cyst<br>2. Umbilical abscess<br>3. Umbilical hernia<br>4. Omphalitis<br>5. Periumbilical cellulitis<br>6. Infected epidermoid cyst<br>7. Ectopic endometriosis<br>8. Intra-	1. Abcess of umbilicus<br>2. Subcutaneous abscess<br>3. Infectious endcardiitis<br>4. Cutaneous Mycobacterial Infections<br>5. Pelvic inflammatory disease<br>6. Sister Mary Joseph's nodule

		10. Crohn’s disease	abdominal abscess<br>9. Diverticulitis<br>10. Pelvic inflammatory disease (PID)	
30	Drug-induced liver injury	1. Drug-induced liver injury (DILI)<br>2. Hepatitis E<br>3. Alcoholic liver disease<br>4. Primary biliary cholangitis (PBC)<br>5. Primary sclerosing cholangitis	1. Drug-induced liver injury (DILI)<br>2. Autoimmune hepatitis<br>3. Primary biliary cholangitis (PBC)<br>4. Primary sclerosing cholangitis (PSC)	1. Drug-induced jaundice<br>2. Liver tuberculosis<br>3. Drug-induced hemolytic anemia<br>4. Autoimmune hemolytic anemia

		(PSC)<br>6. Wilson’s disease<br>7. Autoimmune hepatitis<br>8. Acute liver failure<br>9. Cholangiocarcinoma<br>10. Hemochromatosis	5. Gilbert's syndrome<br>6. Hemolytic anemia<br>7. Alcoholic liver disease<br>8. Non-alcoholic fatty liver disease (NAFLD)<br>9. Hepatic steatosis<br>10. Cholestatic liver disease	
31	Behçet’s disease	1. Behçet’s disease<br>2. Sarcoidosis<br>3. Reactive	1. Behçet's disease<br>2. Reactive arthritis	1. Behçet's disease<br>2. Psoriatic arthritis

		arthritis<br>4. Psoriatic arthritis<br>5. Rheumatoid arthritis<br>6. Systemic lupus erythematosus (SLE)<br>7. Infectious arthritis<br>8. Vasculitis<br>9. Ankylosing spondylitis<br>10. Gout	3. Sarcoidosis<br>4. Ankylosing spondylitis<br>5. Inflammatory bowel disease (IBD)<br>6. Systemic lupus erythematosus (SLE)<br>7. Rheumatoid arthritis<br>8. Psoriatic arthritis<br>9. Lyme disease<br>10. Tuberculosis (TB)	3. Reactive arthritis<br>4. Cogan's syndrome<br>5. Adult onset Still's diesase<br>6. Salmonella infection
32	Rickettsiosis or	1. Rickettsiosis	1. Rickettsiosis	1. Tsutsugamush

leptospirosis	2. Hepatitis<br>3. Urinary tract infection<br>4. Sepsis<br>5. Thrombocytopenia<br>6. Acute kidney injury<br>7. Gastrointestinal bleeding<br>8. Drug-induced liver injury<br>9. Dengue fever<br>10. Leptospirosis	2. Viral infections<br>3. Bacterial infections<br>4. Tick-borne illnesses<br>5. Drug-induced liver injury<br>6. Autoimmune diseases<br>7. Chikungunya virus<br>8. Acute kidney injury<br>9. Hemophagocytic lymphohistiocytosis (HLH)<br>10. Malaria	i disease<br>2. Japanese spotted fever<br>3. Severe fever with thrombocytopenia syndrome<br>4. Tick-borne Relapsing Fever<br>5. Dengue fever<br>6. Zika fever<br>7. Infectious endcardiitis<br>8. Rapidly progressive glomerulonephritis

				9. Hepatitis A<br>10. Hepatitis E
33	Drug-induced eosinophilia	1. Clozapine-induced eosinophilia and systemic symptoms (CESS) or Drug Reaction with Eosinophilia and Systemic Symptoms (DRESS) syndrome<br>2. Clozapine-induced neutropenia	1. Clozapine-induced agranulocytosis or leukocytosis<br>2. Lithium toxicity<br>3. Infection<br>4. Drug-induced liver injury<br>5. Nephritis or nephrotic syndrome<br>6. Eosinophilic pneumonia<br>7. Pleuritis or pericarditis	1. drug fever<br>2. drug induced acute kidney injury<br>3. drug induced hypereosinophilia<br>4. Drug-induced intestinal pneumonia<br>5. aspiration pneumonia<br>6. hypercalcemia<br>7. bacteremia<br>8. infective endocarditis

		3. Clozapine-induced agranulocytosis<br>4. Infectious mononucleosis<br>5. Acute bronchitis<br>6. Pneumonia<br>7. Acute pyelonephritis<br>8. Acute hepatitis<br>9. Acute renal failure<br>10. Acute pancreatitis	8. Systemic inflammatory response syndrome (SIRS) or sepsis<br>9. Drug-induced hypersensitivity syndrome (DIHS)<br>10. Autoimmune or vasculitis disorders	9. organizing pneumonia
34	Brachiocephali	1. Deep vein	1. Superior vena	1. Pancoast

c vein thrombosis	thrombosis (DVT)<br>2. Thrombophlebitis<br>3. Superior vena cava (SVC) syndrome<br>4. Metastatic cancer<br>5. Lung cancer<br>6. Squamous cell carcinoma<br>7. Infection<br>8. Thrombosis<br>9. Malignancy-associated thrombosis<br>10. Pulmonary	cava syndrome (SVCS)<br>2. Thrombophlebitis<br>3. Recurrent/metastatic squamous cell carcinoma<br>4. Infection<br>5. Deep vein thrombosis (DVT)<br>6. Pulmonary infection<br>7. Lymphadenopathy<br>8. Paraneoplastic	tumor secondary to metastasis<br>2. Primary pancoast tumor from lung cancer<br>3. Tuberculoma<br>4. Methothelioma<br>5. Lung abscess<br>6. Lemierre syndrome<br>7. Trousseau syndrome

		embolism (although this has been ruled out in this case)	syndrome<br>9. Venous stasis<br>10. Sepsis	
35	Miliary tuberculosis	1. Bacterial meningitis<br>2. Tuberculous meningitis<br>3. Sepsis<br>4. Encephalitis<br>5. Miliary tuberculosis<br>6. Delirium<br>7. Stroke<br>8. Brain abscess<br>9. Hepatic encephalopath	1. Tuberculous meningitis<br>2. Bacterial meningitis<br>3. Viral meningitis<br>4. Encephalitis<br>5. Sepsis<br>6. Pneumonia<br>7. Urinary tract infection (UTI)<br>8. Endocarditis<br>9. Brain abscess	1. Tuberculous meningitis<br>2. Encephalopathy(due to imipenem + cilastatin)<br>3. Hypercalcemia (due to Tb)<br>4. Carcinomatous meningitis<br>5. Nonconvulsive status epilepticus

		y<br>10. Metastatic cancer	10. Electrolyte imbalance	6. Myxedema coma
36	Vertebral osteomyelitis	1. Spinal osteomyelitis<br>2. Discitis<br>3. Psoas abscess<br>4. Urinary tract infection (UTI)<br>5. Bacteremia<br>6. Hepatitis B<br>7. Compression fractures<br>8. Methicillin-sensitive Staphylococcus aureus	1. Vertebral osteomyelitis<br>2. Spinal epidural abscess<br>3. Discitis<br>4. Psoas muscle abscess<br>5. Pyelonephritis<br>6. Urinary tract infection (UTI)<br>7. Retroperitoneal abscess<br>8. Prostatitis<br>9. Septic arthritis<br>10. Malignancy	1. Acute infective endocarditis<br>2. Vertebral osteomyelitis and dischitis

		(MSSA) infection<br>9. Sepsis<br>10. Dehydration		
37	Vibrio cincinnatiensis infection	1. Vibrio cincinnatiensis infection<br>2. Pseudomonas aeruginosa infection<br>3. Septicemia<br>4. Cellulitis<br>5. Heart failure exacerbation<br>6. Pleural effusion<br>7. Ascites<br>8. Bacteremia	1. Bacteremia<br>2. Skin and soft tissue infection (cellulitis)<br>3. Congestive heart failure<br>4. Sepsis<br>5. Pneumonia<br>6. Diabetic complications<br>7. Liver cirrhosis<br>8. Kidney dysfunction<br>9. Deep vein	1. Acute heart failure caused by Vibrio cincinnatiensis bacteremia<br>2. Cellulitis caused by Vibrio cincinnatiensis and Pseudomonas aeruginosa<br>3. Acute septic cardiomyolitis

		9. Diabetes complications<br>10. Sepsis	thrombosis (DVT)<br>10. Endocarditis	caused by Vibrio cincinnatiensis bacteremia<br>4. infective endocarditis<br>5. diabetic cardiomyolitis<br>6. Takotsubo cardiomyolitis<br>7. perivalvular abscess<br>8. pulmonary embolism<br>9. septic pulmonary embolism
38	Pancreaticople	1. Pancreatic	1. Pancreaticople	1. Pancreatic

ural fistula	cancer<br>2. Pulmonary embolism<br>3. Pleural effusion<br>4. Acute myocardial infarction<br>5. Pneumothorax<br>6. Cardiac tamponade<br>7. Aortic dissection<br>8. Pulmonary fibrosis<br>9. Lung cancer<br>10. Bronchitis	ural fistula<br>2. Pleural effusion due to chronic pancreatitis<br>3. Pneumonia<br>4. Pulmonary embolism<br>5. Heart failure<br>6. Lung cancer<br>7. Tuberculosis<br>8. Atelectasis<br>9. Pulmonary edema<br>10. Chronic obstructive pulmonary disease	pleural effusion caused by rupture of the pancreatic pseudocyst<br>2. Pancreatic pleural effusion from pancreatic cancer<br>3. Pancreatic pleural effusion from chronic pancreatitis<br>4. Pancreatic pleural

			(COPD)	effusion from intraductal papillary mucinous neoplasm
39	Cerebral venous thrombosis	1. Cerebral venous sinus thrombosis (CVST)<br>2. Meningitis<br>3. Subarachnoid hemorrhage (SAH)<br>4. Brain tumor<br>5. Migraine headache<br>6. Cluster headache	1. Cerebral venous sinus thrombosis (CVST)<br>2. Migraine<br>3. Tension headache<br>4. Idiopathic intracranial hypertension (IIH)<br>5. Brain tumor<br>6. Infection or	1. superior sagittal sinus and left lateral sinus thrombosis<br>2. subarachnoid hemorrhage<br>3. crowned dence syndrome<br>4. migraine<br>5. cervical spondylosis

		7. Intracranial hypertension<br>8. Encephalitis<br>9. Chiari malformation<br>10. Arteriovenous malformation (AVM)	inflammation (e.g., meningitis, encephalitis)<br>7. Subarachnoid hemorrhage<br>8. Sinusitis<br>9. Medication overuse headache<br>10. Cluster headache	6. cluster headache<br>7. tenshion headache<br>8. great occipital neuralgia
40	Acute cytomegalovirus infection	1. Viral infection<br>2. Infectious mononucleosis<br>3. Bacterial infection	1. Cytomegalovirus (CMV) infection<br>2. Epstein-Barr virus (EBV) infection	1. Drug-induced hypersensitivity syndrome<br>2. Infective mononucleosis like

		4. Thyroiditis<br>5. Asthma exacerbation<br>6. Drug reaction<br>7. Urinary tract infection<br>8. Septicemia<br>9. Non-specific fever<br>10. COVID-19	3. Influenza<br>4. Atypical pneumonia<br>5. Drug-induced fever<br>6. Viral hepatitis<br>7. Autoimmune disease<br>8. Bacterial infection<br>9. Vasculitis<br>10. Tick-borne illness	syndrome due to CMV reactivation<br>3. Infective mononucleosis like syndrome due to HIV<br>4. Modified measles<br>5. Rubella<br>6. Toxoplasmosis<br>7. Mycoplasma pneumoniae infection
41	Fitz-Hugh-Curtis syndrome	1. Chlamydia infection<br>2. Pelvic	1. Pelvic inflammatory disease (PID)	1. Fitz-Hugh-Curtis syndrome

		inflammatory disease (PID)<br>3. Gallbladder disease<br>4. Hepatitis<br>5. Appendicitis<br>6. Ectopic pregnancy<br>7. Ovarian cyst<br>8. Kidney infection or stone<br>9. Pancreatitis<br>10. Musculoskeletal pain	2. Ectopic pregnancy<br>3. Appendicitis<br>4. Cholecystitis<br>5. Pyelonephritis<br>6. Peritonitis<br>7. Pleurisy<br>8. Liver abscess<br>9. Pneumonia<br>10. Musculoskeletal pain	2. Pulmonary embolism
42	Aortic intramural haematomaac	1. Aortic Dissection<br>2. Aortic	1. Aortic dissection<br>2. Intramural	1. aortopulmonary connective sheath

ute aortic syndrome<br>aortic dissection	Aneurysm<br>3. Myocardial Infarction<br>4. Pulmonary Embolism<br>5. Acute Pancreatitis<br>6. Peptic Ulcer Disease<br>7. Aortic Rupture<br>8. Pneumothorax<br>9. Spinal Cord Compression<br>10. Acute Cholecystitis	hematoma<br>3. Aortic aneurysm<br>4. Thoracic aortic injury<br>5. Acute coronary syndrome (ACS)<br>6. Unstable angina<br>7. Myocardial infarction<br>8. Pulmonary embolism<br>9. Musculoskeletal pain<br>10. Pericarditis	hematoma<br>2. aortic dissection<br>3. Acute coronary syndrome<br>4. Takayasu arthritis<br>5. giant cell arthritis<br>6. aortic aneurythm<br>7. Microvascular stenosis

43	A seroma in the gallbladder fossa	1. Bile leak<br>2. Gallstone ileus<br>3. Gallbladder cancer<br>4. Postoperative infection<br>5. Pancreatitis<br>6. Cholangitis<br>7. Acute hepatitis<br>8. Liver abscess<br>9. Duodenitis<br>10. Perforated peptic ulcer disease	1. Postoperative infection<br>2. Biliary leak<br>3. Abscess<br>4. Bile duct injury<br>5. Retained gallstones<br>6. Biliary obstruction<br>7. Bowel obstruction<br>8. Cholangitis<br>9. Gastroenteritis<br>10. Pancreatitis	1. Vasculitis of the arteries feeding the gallbladder fossa<br>2. gallbladder fossa abscess<br>3. cholangiocarcinoma<br>4. cholangitis<br>5. IgG4 related cholangitis<br>6. ANCA associated vasculitis<br>7. bile duct sarcoidosis
44	Idiopathic	1. Meningitis	1. Central	1. Giant cell

hypertrophic pachymeningitis	2. Encephalitis<br>3. Cerebral edema<br>4. Granulomatosis with polyangiitis (GPA)<br>5. Sarcoidosis<br>6. Lymphoma<br>7. Idiopathic intracranial hypertension (IIH)<br>8. Multiple sclerosis (MS)<br>9. Lupus cerebritis<br>10. Neurosarcoido	nervous system (CNS) vasculitis<br>2. Giant cell arteritis (GCA)<br>3. Infections such as meningitis or encephalitis<br>4. Neurosarcoidosis<br>5. Autoimmune encephalitis<br>6. Chronic meningitis<br>7. Paraneoplastic syndromes<br>8. Idiopathic hypertrophic	arteritis with PMR<br>2. Central nervous system lymphoma<br>3. HTLV-1 infection<br>4. Glucocorticoid withdrawal

		sis	pachymeningitis (IHPM)<br>9. Lymphomatous meningitis<br>10. Intracranial hypotension	
45	Achenbach syndrome.	1. Trauma<br>2. Raynaud’s disease<br>3. Thrombosis<br>4. Aneurysm<br>5. Vasculitis<br>6. Infection<br>7. Gout<br>8. Lupus<br>9. Scleroderma<br>10. Cancer	1. Traumatic injury<br>2. Insect bite or sting<br>3. Spontaneous hematoma<br>4. Paronychia<br>5. Raynaud's phenomenon<br>6. Vasculitis<br>7. Acute gout<br>8. Tendonitis	1. Achenbach's syndrome<br>2. Trauma of the finger<br>3. Subcutaneous bleeding with wWF deficiency

			9. Cellulitis:<br>10. Foreign body reaction	
46	Acute pulmonary embolism	1. Pulmonary Embolism<br>2. Heart failure<br>3. Aortic stenosis<br>4. Arrhythmia<br>5. Pneumonia<br>6. Chronic obstructive pulmonary disease (COPD)<br>7. Asthma<br>8. Hypotension<br>9. Anemia<br>10. Dehydration	1. Pulmonary embolism<br>2. Congestive heart failure<br>3. Cardiac arrhythmia<br>4. Dehydration or electrolyte imbalance<br>5. Vasovagal syncope<br>6. Orthostatic hypotension<br>7. Anemia<br>8. Anxiety or	1. Pulmonary embolism<br>2. syncope induced by vasovagal reflexion<br>3. Orthostatic hypotention<br>4. sycope induced by arrythmia (i.e. complete AV block)<br>5. cerebrospinal fluid

			panic attack<br>9. Chronic obstructive pulmonary disease (COPD)<br>10. Pneumonia	hypovolemia<br>6. subcravian steel syndrome<br>7. Vagus nerve reflex caused by arterial sinus compression
47	Colorectal carcinoma	1. Spinal Epidural Abscess<br>2. Spinal Cord Compression<br>3. Osteomyelitis (infection of the bone)<br>4. Septicemia	1. Metastatic cancer<br>2. Infectious spondylodiscitis<br>3. Tuberculosis<br>4. Bacterial endocarditis<br>5. Brucellosis	1. multiple bone metastasis caused by Borrmann type II tumor<br>2. multiple bone metastasis caused by prostate

(bloodstream infection)

5. Metastatic cancer to the bone
6. Multiple myeloma (cancer of plasma cells)
7. Lymphoma (cancer of the lymphatic system)
8. Malignant bone tumor (e.g., osteosarcoma)
9. Neurological

6. Multiple myeloma
7. Lymphoma
8. Osteomyelitis
9. Sarcoidosis
10. Vasculitis

cancer

3. Erdheim-Chester disease
4. TAFRO syndrome
5. antibiotics induced lower limbs paralysis
6. post laminectomy abscess
7. bacterial osteomyolitis
8. osteomyolitis caused by tuberculosis
9. osteomyolitis

		disorder (e.g., multiple sclerosis)<br>10. Infective endocarditis (infection of the heart valves)		caused by non-tuberculosis mycrobacterium
48	Ramsay Hunt syndrome	1. Vestibular Neuritis<br>2. Benign Paroxysmal Positional Vertigo (BPPV)<br>3. Meniere’s Disease<br>4. Cervical	1. Labyrinthitis<br>2. Vestibular neuritis<br>3. Meniere's disease<br>4. Benign paroxysmal positional vertigo (BPPV)<br>5. Ramsay Hunt	1. Ramsay Hunt syndrome<br>2. Cholesteatoma with chronic otitis media<br>3. Mycoplasma infecton and Guillain-Barre syndrome<br>4. Vasculitis

		Vertigo<br>5. Labyrinthitis<br>6. Stroke<br>7. Acoustic Neuroma<br>8. Ramsay Hunt Syndrome<br>9. Multiple Sclerosis (MS)<br>10. Migraine-Associated Vertigo	syndrome<br>6. Stroke or transient ischemic attack (TIA)<br>7. Cervical vertigo<br>8. Medication side effects<br>9. Acoustic neuroma<br>10. Central nervous system (CNS) infections	5. Vertebrobasilar insufficiency<br>6. Subclavian artery steal syndrome
49	Food poisoning	1. Diabetic neuropathy<br>2. Peripheral	1. Diabetic neuropathy<br>2. Mushroom	1. Mushrooms poisning<br>2. Tetanus

neuropathy<br>3. Toxic neuropathy<br>4. Guillain-Barre Syndrome<br>5. Charcot-Marie-Tooth disease<br>6. Lyme disease<br>7. Vitamin B12 deficiency<br>8. Multiple sclerosis<br>9. Hypothyroidism<br>10. Mushroom toxicity	poisoning<br>3. Contact dermatitis<br>4. Peripheral artery disease (PAD)<br>5. Peripheral neuropathy<br>6. Complex regional pain syndrome (CRPS)<br>7. Guillain-Barre syndrome<br>8. Raynaud's phenomenon<br>9. Tarsal tunnel syndrome	3. Guillain-Barré Syndrome<br>4. Restless legs syndrome<br>5. Diabetic neuropathy<br>6. Parkinson's disease<br>7. Pellagra<br>8. Cannabis intoxication

			(foot) and carpal tunnel syndrome (hand)<br>10. Infection	
50	Vitamin B_{12} deficiency	1. Pernicious anemia<br>2. Thrombotic thrombocytopenic purpura (TTP)<br>3. Disseminated intravascular coagulation (DIC)<br>4. Sepsis<br>5. Acute kidney injury (AKI)	1. Pernicious anemia<br>2. Thrombotic thrombocytopenic purpura (TTP)<br>3. Hemolytic uremic syndrome (HUS)<br>4. Disseminated intravascular coagulation	1. Subacute combined spinal cord degeneration<br>2. Myelodysplastic syndromes<br>3. Primary central nervous system lymphoma<br>4. Tuberculous meningitis

		6. Liver failure<br>7. Iron-deficiency anemia<br>8. Hypertensive heart disease<br>9. Multiple myeloma<br>10. Stroke	(DIC)<br>5. Autoimmune hemolytic anemia<br>6. Chronic kidney disease<br>7. Liver disease<br>8. Hypothyroidism<br>9. Vitamin B12 deficiency<br>10. Pulmonary infection	5. Neurosyphilis<br>6. COVID-19
51	Osmotic demyelination syndrome	1. Diabetic hyperosmolar syndrome<br>2. Diabetic ketoacidosis	1. Hyperosmolar Hyperglycemic State (HHS)<br>2. Hypoglycemia<br>3. Diabetic	1. ODS: Osmotic Demyelination Syndrome caused by hyperclycemia

3. Cerebral edema
4. Stroke
5. Hypoglycemia
6. Hypertensive encephalopathy
7. Wernicke encephalopathy
8. Multiple sclerosis
9. Brain tumor
10. Encephalitis

neuropathy

4. Hypertensive encephalopathy
5. Cerebrovascular accident (stroke)
6. Central pontine myelinolysis (osmotic demyelination syndrome)
7. Diabetic ketoacidosis (DKA)
8. Encephalitis
9. Seizures

2. multiple brain stroke
3. hyperosmoler hypergycemic syndrome
4. infective endocarditis
5. hypoglycemic encephalopathy
6. septic encepharopathy
7. hyperactive derilium
8. schixophrenia
9. Acute viral encepharopat

			10. Meningitis	hy
52	Intramural haematoma of the oesophagus	1. Oesophageal perforation<br>2. Oesophageal hematoma<br>3. Gastrointestinal bleeding<br>4. Peptic ulcer disease<br>5. Acute coronary syndrome<br>6. Myocardial infarction<br>7. Gastritis<br>8. Pancreatitis<br>9. Cholecystitis<br>10. Diverticulitis	1. Spontaneous intramural hematoma of the esophagus (SIHE)<br>2. Esophageal rupture<br>3. Acute cholecystitis<br>4. Mallory-Weiss tear<br>5. Peptic ulcer disease<br>6. Gastroesophageal reflux disease (GERD)	1. aortic dissection<br>2. concentric intramural haematoma<br>3. coronary artery dissection<br>4. coronary artery aneurythm<br>5. esophageal rupture<br>6. Rupture of an esophageal varix<br>7. mallory-wise

	7. Acute pancreatitis<br>8. Acute coronary syndrome (ACS)<br>9. Boerhaave syndrome<br>10. Gastritis	syndrome<br>8. Bronchial artery rupture

Notes: the number was same as the Table S1 of Multimedia Appendix 1.

Abbreviations: ChatGPT-3, third generation generative pre-trained transformer; ChatGPT-4, fourth generation generative pre-trained transformer.
